# Latent Class Analysis with Arbitrary-Distribution Responses

**DOI:** 10.3390/e27080866

**Published:** 2025-08-14

**Authors:** Huan Qing, Xiaofei Xu

**Affiliations:** 1 School of Economics and Finance, Chongqing University of Technology, Chongqing 400054, China; qinghuan@cqut.edu.cn; 2 School of Mathematics and Statistics, Wuhan University, Wuhan 430070, China

**Keywords:** categorical data, latent class model, spectral method, SVD, arbitrary-distribution responses

## Abstract

The latent class model has been proposed as a powerful tool in understanding human behavior for various fields such as social, psychological, behavioral, and biological sciences. However, one important limitation of the latent class model is that it is primarily applied to data with binary responses or categorical responses, making it fail to model real-world data with continuous or negative responses. In many applications, ignoring the weights throws out a lot of potentially valuable information contained in the weights. To address this limitation, we propose a novel generative model, the arbitrary-distribution latent class model (adLCM). Our model enables the generation of data’s response matrix from an arbitrary distribution with a latent class structure. When compared to the latent class model, our adLCM is both more realistic and general. To our knowledge, our adLCM is the first model for latent class analysis with any real-valued responses, including continuous, negative, and signed values, thereby extending the classical latent class model beyond its traditional limitation to binary or categorical outcomes. We investigate the identifiability of the model and propose an efficient algorithm for estimating the latent classes and other model parameters. We show that the proposed algorithm enjoys consistent estimation. The performance of our algorithm is evaluated using both computer-generated data and real-world personality test data.

## 1. Introduction

The latent class model (LCM) [1,2,3] is a powerful tool for categorical data, with many applications across various areas such as social, psychological, behavioral, and biological sciences. These applications include movie rating [4,5], psychiatric evaluation [6,7,8,9], educational assessments [10], political surveys [11,12,13,14], transport economics personal interviews [15], and disease etiology detection [16,17,18]. In categorical data, subjects (individuals) typically respond to several items (questions). LCM is a theoretical model that categorizes subjects into disjoint groups, known as latent classes, according to their response pattern to a collection of categorical items. Latent classes assist researchers in better understanding human behaviors. For example, in movie rating, latent classes may represent different groups of users with an affinity for certain movie themes; in psychological tests, latent classes may represent different types of personalities. In educational assessments, latent classes may indicate different levels of abilities. In political surveys, latent classes may represent distinct types of political ideologies. In transport economics personal interviews, each latent class stands for a partition of the population. In disease etiology detection, latent classes may represent different disease categories. To infer latent classes for categorical data generated from LCM, various approaches have been developed in recent years, including maximum likelihood estimation techniques [19,20,21,22,23], nonnegative matrix factorization (NMF) [24], tensor-based methods [25,26], and spectral clustering approaches [27,28,29].

To mathematically describe categorical data, let *R* be the *N*-by-*J* observed response matrix such that R(i,j) represents subject *i*’s response to item *j*, where *N* denotes the number of subjects and *J* denotes the number of items. For LCM, many researchers focus on binary choice data where elements of the observed response matrix *R* only take 0 or 1 [10,16,26,30,31,32,33,34,35,36,37]. LCM models the binary (or categorical) response matrix by generating its elements from a Bernoulli (or Binomial) distribution. Binary responses can be agree/disagree responses in psychiatric evaluation, correct/wrong responses in educational assessments, and presence/absence of symptoms in disease etiology detection. For real-world categorical data from various online personality tests in the link https://openpsychometrics.org/_rawdata/ (accessed on 11 August 2025), the ranges of most categorical responses are {0,1,2,…,m}, where *m* is an integer like 2, 5, and 10. However, categorical data is more than binary or categorical responses. Categorical data with negative or continuous responses is also commonly encountered in the real world, and ignoring such weighted data may lose potentially meaningful information [38]. For example, in the buyer–seller rating e-commerce data [39], elements of the observed response matrix take values of {−1,0,1} (for convenience, we call such *R* as a signed response matrix in this paper) since sellers are rated by users by applying three levels of rating, “Positive”, “Neutral”, and “Negative”. In the users–jokes rating categorical data Jester 100 [40], with the data source link: https://eigentaste.berkeley.edu/dataset/archive/ (accessed on 11 August 2025), all responses (i.e., ratings) are continuous numbers in the range [−10,10]. The aforementioned data cannot be generated from a Bernoulli or Binomial distribution. On the other hand, a substantial body of work has been developed to address polytomous responses or continuous responses, such as profile analysis (LPA) and factor mixture models (FMMs) [41,42,43,44,45,46,47], while their restrictive Gaussian or factorial assumptions limit their applicability to more general response types with irregular scales, such as real-valued ratings or signed categorical scores as aforementioned. Therefore, it is desirable to develop a more flexible model for data with arbitrary-distribution responses. With this motivation, our key contributions to the literature of latent class analysis are summarized as follows.

- Model. We propose a novel, identifiable, and generative statistical model, the arbitrary-distribution latent class model (adLCM), for data with arbitrary-distribution responses, where the responses can be continuous or negative values. Our adLCM allows the elements of an observed response matrix *R* to be generated from any distribution provided that the population version of *R* under adLCM enjoys a latent class structure. For example, our adLCM allows *R* to be generated from Bernoulli, Normal, Poisson, Binomial, Uniform, and Exponential distributions, etc. By considering a specifically designed discrete distribution, our adLCM can also model signed response matrices. For details, please refer to Examples 1–7.
- Algorithm. We develop an easy-to-implement algorithm, spectral clustering with K-means (SCK), to infer latent classes for arbitrary-distribution response matrices generated from arbitrary distribution under the proposed model. Our algorithm is designed based on a combination of two popular techniques: the singular value decomposition (SVD) and the K-means algorithm.
- Theoretical property. We build a theoretical framework to show that SCK enjoys consistent estimation under adLCM. We also provide Examples 1–7 to show that the theoretical performance of the proposed algorithm can be different when the observed response matrices *R* are generated from different distributions under the proposed model.
- Empirical validation. We conduct extensive simulations to validate our theoretical insights. Additionally, we apply our SCK approach to two real-world personality test datasets with meaningful interpretations.

The remainder of this paper is organized as follows. Section 2 offers a comprehensive review of related works. Section 3 describes the model. Section 4 details the algorithm. Section 5 establishes the consistency results and provides examples for further analysis. Section 6 contains numerical studies that verify our theoretical findings and examine the performance of the proposed method. Section 7 demonstrates the proposed method using two real-world datasets. Section 8 concludes the paper with a brief discussion of contributions and future work.

The following notations will be used throughout the paper. For any positive integer *m*, let [m] and $I_{m\times m}$ be [m]:={1,2,…,m} and the m×m identity matrix, respectively. For any vector *x* and any q>0, ${∥x∥}_{q}$ denotes *x*’s $l_{q}$-norm. For any matrix *M*, $M^{\prime }$ denotes its transpose, ∥M∥ denotes its spectral norm, ${∥M∥}_{F}$ denotes its Frobenius norm, rank(M) denotes its rank, $\sigma_{i}\left(M\right)$ denotes its *i*-th largest singular value, $\lambda_{i}\left(M\right)$ denotes its *i*-th largest eigenvalue ordered by magnitude, M(i,:) denotes its *i*-th row, and M(:,j) denotes its *j*-th column. Let R and N be the set of real numbers and nonnegative integers, respectively. For any random variable *X*, E(X) and P(X=a) are the expectation and the probability that *X* equals to *a*, respectively. Let $M_{m,K}$ be the collection of all m×K matrices where each row has only one 1 and all others 0.

## 2. Related Literature

Categorical data have been widely collected in various fields ranging from social and psychological research to political and transportation sciences [4,5,6,7,8,9,10,11,12,13,14,15,16,17,18]. The latent class model (LCM) has long been a cornerstone for modeling unobserved heterogeneity in categorical data that are not directly measurable [48,49]. Since [48] provided one of the earliest formal frameworks for latent structure models in sociology, LCM has been widely applied in psychology and medicine for identifying subgroups of individuals with similar symptom or trait profiles. For example, Meyer et al. [6] applied latent classes to represent different personality or clinical profiles in psychometric assessments. In political science, LCM is used to analyze voting patterns and public opinion. For instance, Poole [11] applied an unfolding approach to cluster legislators based on their yes/no voting patterns. Wu et al. [18] developed a nested latent class model that distinguished pathogen combinations causing childhood pneumonia in epidemiology. LCM is also employed in health outcomes research [16] or to cluster clinical profiles [17].

For the classical LCM, the responses of categorical data are often assumed to be binary or categorical with nonnegative integers, such as the binary responses of agree/disagree (or yes/no) or ordinal categories in certain surveys [26,30,31,32,33,34,35,36,37]. Compared to traditional clustering methods like K-means and K-modes, LCM provides a model-based clustering approach with the selection of the clusters based on rigorous statistical tests [28]. Various studies in the literature have extended and refined latent class techniques in diverse directions. For example, some studies focus on the fundamental concern of identifiability in LCM to evaluate the feasibility of recovering model parameters and latent classes [37,50]. Gyllenberg et al. [51] stated that the LCM with binary responses is not identifiable; this problem has received considerable attention in [34,37] for extended LCMs. Another extension is the grade-of-membership model (GoM), which allows individuals to have partial membership in multiple classes rather than a single discrete class [52]. Qing [28] detected mixed memberships in categorical data with polytomous responses based on the GoM model and derived theoretical guarantees as well; see also [53,54,55]. Meanwhile, the estimation of LCM has also spurred extensive methodological innovation. For example, the maximum likelihood estimates are typically used via the expectation–maximization algorithm to iteratively refine class membership and parameter estimates [22,23,56]. Clinton et al. [12] considered Bayesian inference using MCMC techniques to analyze roll-call voting data, treating the class memberships as parameters to be sampled, see also [57,58,59] for more Bayesian reference. Recently, the tensor-based [25,26] and spectral-based algorithms [27,28] have also been popular to infer the latent memberships in the LCM framework.

As mentioned earlier, while classical latent class models are powerful for categorical data, they have a significant limitation as they typically assume binary or ordinal categories for the response. The literature has stated that the data could have arbitrary distribution with general responses such as negative or continuous values; ignoring this information may lead to a misunderstanding of the latent class structures [38]. Various methods have been proposed to address polytomous or continuous response data, such as latent profile analysis (LPA) and factor mixture models (FMM) [41,42,43,44,45,46,47]. However, for more general response types—such as the Jester joke data with ratings continuously varying within [−10,10] [40], e-commerce data with scores in the range {−1,0,1} [39], or the Advogato trust data with relationship values of {0.6,0.8,1}[60]—the classical LCM, LPA, and FMM are not applicable, due to LPA’s Gaussian assumption and FMM’s factorial assumption. To address this limitation, this paper proposes an arbitrary-distribution latent class model to enable the generation of data’s response matrix from an arbitrary distribution with a latent class structure. Hence, it allows any real-valued responses, including continuous, negative, and signed values, not merely limited to sampling weights, and thereby extends the classical LCM beyond its traditional response limitation.

In recent years, a noteworthy trend in latent structure learning is the use of spectral and other matrix/tensor-based methods to learn latent class structures as alternatives to traditional likelihood-based inference. Spectral algorithms offer an alternative by leveraging linear algebra (eigen-decomposition, singular value decomposition) or tensors of the data with theoretical soundness and ease of implementation [25]. In the context of network community detection—a problem analogous to finding latent classes of nodes—spectral clustering on graph Laplacians became a dominant approach for diverse types of networks [27,61,62,63]. Such algorithms have been extended to mixed-membership stochastic block models to allow overlapping communities [64,65]. In the latent class analysis realm, spectral algorithms have recently been developed to consistently estimate LCM parameters. For example, Chen and Gu [27] applied a spectral method for identifying GoM parameters from binary response data. Similarly, Anandkumar et al. [25] described tensor decomposition techniques to solve latent variable models via the factorization of third-order moments. These spectral methods, surveyed comprehensively by [66], achieve sound estimation and asymptotic consistency. Though recent advances extend spectral learning to polytomous categorical data, most existing applications still focus on binary settings. This paper focuses on the spectral method for arbitrary-distribution latent class modeling, and develops an easy-to-implement spectral clustering algorithm based on singular value decomposition (SVD) and the K-means algorithm.

## 3. Arbitrary-Distribution Latent Class Model

Unlike most researchers that focus on binary responses, in our arbitrary-distribution response setting in this paper, all elements of the observed response matrix *R* are allowed to be any real value, i.e., $R\in R^{N\times J}$.

Consider categorical data with *N* subjects and *J* items, where the *N* subjects belong to *K* disjoint extreme latent profiles (also known as latent classes). Throughout this paper, the number of classes *K* is assumed to be a known integer. To describe the membership of each subject, we let *Z* be a N×K matrix such that Z(i,k) is 1 if subject *i* belongs to the *k*-th extreme latent profile and Z(i,k) is 0 otherwise. Call *Z* the classification matrix in this paper. For each subject i∈[N], it is assumed to belong to a single extreme latent profile. For convenience, define *ℓ* as an *N*-by-1 vector whose *i*-th entry ℓ(i) is *k* if the *i*-th subject belongs to the *k*-th extreme latent profile for i∈[N]. Thus, for subject i∈[N], we have Z(i,ℓ(i))=1 and the other (K−1) entries of the K×1 classification vector Z(i,:) are 0.

Introduce the J×K item parameter matrix $\Theta \in R^{J\times K}$. For k∈[K], our arbitrary-distribution latent class model (adLCM) assumes that Θ(j,k) collects the conditional-response expectation for the response of the *i*-th subject to the *j*-th item under arbitrary distribution F provided that subject *i* belongs to the *k*-th extreme latent profile. Specifically, for i∈[N],j∈[J], given the classification vector Z(i,:) of subject *i* and the item parameter matrix Θ, our adLCM assumes that for arbitrary distribution F, the conditional response expectation of the *i*-th subject to the *j*-th item is

(1) $$\begin{array}{c} E\left(R\left(i,j\right)|Z\left(i,:\right),\Theta \right)=\sum_{k=1}^{K}Z\left(i,k\right)\Theta \left(j,k\right)\equiv \Theta \left(j,\ell \left(i\right)\right). \end{array}$$

Based on Equation (1), our adLCM can be simplified as follows.

**Definition** **1.** *Let $R\in R^{N\times J}$ denote the observed response matrix. Let $Z\in M_{N,K}$ be the classification matrix and $\Theta \in R^{J\times K}$ be the item parameter matrix. For i∈[N], j∈[J], our arbitrary-distribution latent class model (adLCM) assumes that for an arbitrary distribution F, R(i,j) are independent random variables generated from the distribution F and the expectation of R(i,j) under the distribution F should satisfy the following formula:*

(2) $$\begin{array}{c} E\left(R\left(i,j\right)\right)=R_{0}\left(i,j\right),\mathrm{where}\;R_{0}:=Z\Theta^{\prime }. \end{array}$$

Definition 1 says that adLCM is determined by the classification matrix *Z*, the item parameter matrix Θ, and the distribution F. For brevity, we denote adLCM by adLCM(Z,Θ,F). Under adLCM, F is allowed to be any distribution as long as Equation (2) is satisfied under F, i.e., adLCM only requires the expectation (i.e., population) response matrix $R_{0}$ of the observed response matrix *R* to be $Z\Theta^{\prime }$ under any distribution F.

**Remark** **1.** *For the case that F is a Bernoulli distribution, all elements of Θ have a range in [0,1], R only contains binary responses (i.e., R(i,j)∈{0,1} for i∈[N], j∈[J] when F is a Bernoulli distribution), and Equation (1) becomes P(R(i,j)=1|Z(i,:),Θ)=Θ(j,ℓ(i)). For this case, adLCM reduces to the LCM model for data with binary responses.*

**Remark** **2.** *It should be noted that Equation (2) does not hold for all distributions. For instance, we cannot set F as a t-distribution because the expectation of a t-distribution is always 0, which cannot capture the latent structure required by adLCM; F cannot be a Cauchy distribution whose expectation does not even exist; F cannot be a Chi-square distribution because the expectation of a Chi-square distribution is its degrees of freedom, which is a fixed positive integer and cannot capture the latent structure required by adLCM. We will provide some examples to demonstrate that Equation (2) can be satisfied for different distributions of F. For details, please refer to Examples 1–7.*

**Remark** **3.** *It should also be noted that the ranges of the observed response matrix R and the item parameter matrix* Θ *depend on distribution F. For example, when F is a Bernoulli distribution, $R\;\in \;{\left\{0,1\right\}}^{N\times J}$ and $\Theta \in {\left[0,1\right]}^{J\times K}$; when F is a Poisson distribution, $R\in N^{N\times J}$ and $\Theta \;\in \;{\left[0,+\infty \right)}^{J\times K}$. If we let F be a Normal distribution, $R\in R^{N\times J}$ and $\Theta \in {\left(-\infty ,+\infty \right)}^{J\times K}$. For details, please refer to Examples 1–7.*

The following proposition shows that adLCM is identifiable as long as there exists at least one subject for every extreme latent profile.

**Proposition** **1** (Identifiability)**.** *Consider the adLCM as in Equation (2): when each extreme latent profile has at least one subject, the model is identifiable: for any other valid parameter set $\left(\tilde{Z},\tilde{\Theta }\right)$, if $\tilde{Z}{\tilde{\Theta }}^{\prime }=Z\Theta^{\prime }$, then (Z,Θ) and $\left(\tilde{Z},\tilde{\Theta }\right)$ are identical up to a permutation of the K extreme latent profiles.*

All proofs of theoretical results developed in this paper are given in the Appendix A. The condition that each extreme latent profile must contain at least one subject means that each extreme latent profile cannot be an empty set and we have rank(Z)=K.

**Remark** **4.** *Note that Z and $\tilde{Z}$ are the same up to a permutation of the K latent classes in Proposition 1. A permutation is acceptable since the equivalence of Z and $\tilde{Z}$ should not rely on how we label each of the K extreme latent profiles. A similar argument holds for the identity of* Θ*; and $\tilde{\Theta }$.*

The observed response matrix *R* along with the ground-truth classification matrix *Z* and the item parameter matrix Θ can be generated using our adLCM as follows: let R(i,j) be a random variable generated by distribution F with expected value $R_{0}\left(i,j\right)$ for i∈[N], j∈[J], where $R_{0}=Z\Theta^{\prime }$ satisfies the latent structure required by adLCM. In latent class analysis, given the observed response matrix *R* generated from adLCM(Z,Θ,F), our goal is to infer the classification matrix *Z* and the item parameter matrix Θ. Proposition 1 ensures that the model parameters *Z* and Θ can be reliably inferred from the observed response matrix *R*. In the following two sections, we will develop a spectral algorithm to fit adLCM and show that this algorithm yields consistent estimation.

## 4. A Spectral Method for Parameter Estimation

In addition to providing a more general model for latent class analysis, we are also interested in estimating the model parameters. In this section, we focus on the parameter estimation problem within the adLCM framework by developing an efficient and easy-to-implement spectral method.

To provide insight into developing an algorithm for the adLCM, we first consider an oracle case where we observe the expectation response matrix $R_{0}$ given in Equation (2). We would like to estimate *Z* and Θ from $R_{0}$. Recall that the item parameter matrix Θ is a *J*-by-*K* matrix; here we let $\mathrm{rank}\left(\Theta \right)=K_{0}$, where $K_{0}$ is a positive integer and it is no larger than *K*. As $R_{0}=Z\Theta^{\prime },\mathrm{rank}\left(Z\right)=K$, and $\mathrm{rank}\left(\Theta \right)=K_{0}\leq K$, we see that $R_{0}$ is a rank-$K_{0}$ matrix. As the number of extreme latent profiles *K* is usually far smaller than the number of subjects *N* and the number of items *J*, the *N*-by-*J* population response matrix $R_{0}$ enjoys a low-dimensional structure. Next, we will demonstrate that we can greatly benefit from the low-dimensional structure of $R_{0}$ when we aim to develop a method to infer model parameters under adLCM.

Let $R_{0}=U\Sigma V^{\prime }$ be the compact singular value decomposition (SVD) of $R_{0}$ such that Σ is a $K_{0}\times K_{0}$ diagonal matrix collecting the $K_{0}$ nonzero singular values of $R_{0}$. Write $\Sigma =\mathrm{diag}(\sigma_{1}\left(R_{0}\right),\sigma_{2}\left(R_{0}\right),\ldots ,\sigma_{K_{0}}\left(R_{0}\right))$. The $N\times K_{0}$ matrix *U* collects the corresponding left singular vectors and it satisfies $U^{\prime }U=I_{K_{0}\times K_{0}}$. Similarly, the $J\times K_{0}$ matrix *V* collects the corresponding right singular vectors and it satisfies $V^{\prime }V=I_{K_{0}\times K_{0}}$. For k∈[K], let $N_{k}$ be the number of subjects that belong to the *k*-th extreme latent profile, i.e., $N_{k}=\sum_{i=1}^{N}Z\left(i,k\right)$. The ensuing lemma constitutes the foundation of our estimation method.

**Lemma** **1.** *Under adLCM(Z,Θ,F), let $R_{0}=U\Sigma V^{\prime }$ be the compact SVD of $R_{0}$. The following statements are true.*

- *The left singular vectors matrix U can be written as*
(3) $$\begin{array}{c} U=ZX, \end{array}$$
*where X is a $K\times K_{0}$ matrix.*
- *U has K distinct rows such that for any two distinct subjects i and $\bar{i}$ that belong to the same extreme latent profile (i.e., $\ell \left(i\right)=\ell \left(\bar{i}\right)$), we have $U\left(i,:\right)=U\left(\bar{i},:\right)$.*
- Θ *can be written as*
  (4) $$\begin{array}{c} \Theta =V\Sigma U^{\prime }Z{\left(Z^{\prime }Z\right)}^{-1}. \end{array}$$
- *Furthermore, when $K_{0}=K$, for all k∈[K],l∈[K], and k≠l, we have*
(5) $$\begin{array}{c} {∥X\left(k,:\right)-X\left(l,:\right)∥}_{F}={\left(N_{k}^{-1}+N_{l}^{-1}\right)}^{1/2}. \end{array}$$

From now on, for the simplicity of our further analysis, we let $K_{0}\equiv K$. Hence, the last statement of Lemma 1 always holds.

The second statement of Lemma 1 indicates that the rows of *U* corresponding to subjects assigned to the same extreme latent profile are identical. This circumstance implies that the application of a clustering algorithm to the rows of *U* can yield an exact reconstruction of the classification matrix *Z* after a permutation of the *K* extreme latent profiles.

In this paper, we adopt the K-means clustering algorithm, an unsupervised learning technique that groups similar data points into *K* clusters. This clustering technique is detailed as follows,

(6) $$\begin{array}{c} \left(\bar{\bar{Z}},\bar{\bar{X}}\right)=\arg\;\min_{\bar{Z}\in M_{N,K},\bar{X}\in R^{K\times K}}{∥\bar{Z}\bar{X}-\bar{U}∥}_{F}^{2}, \end{array}$$

where $\bar{U}$ is any N×K matrix. For convenience, call Equation (6) a “Run K-means algorithm on all rows of $\bar{U}$ with *K* clusters to obtain $\bar{\bar{Z}}$” because we are interested in the classification matrix $\bar{\bar{Z}}$. Let $\bar{U}$ in Equation (6) be *U*; the second statement of Lemma 1 guarantees that $\bar{\bar{Z}}=ZP,\bar{\bar{X}}=P^{\prime }X$, where P is a K×K permutation matrix, i.e., the running K-means algorithm on all rows of *U* exactly recovers *Z* up to a permutation of the *K* extreme latent profiles.

After obtaining *Z* from *U*, Θ can be recovered subsequently by Equation (4). The above analysis suggests the following Algorithm 1, Ideal SCK, where SCK stands for Spectral Clustering with K-means. Ideal SCK returns a permutation of (Z,Θ), which also supports the identifiability of the proposed model as stated in Proposition 1.
**Algorithm 1** Ideal SCK**Require:** The expectation response matrix $R_{0}$ and the number of extreme latent profiles *K*.**Ensure:** A permutation of *Z* and Θ.  1:Obtain $U\Sigma V^{\prime }$, the top *K* SVD of $R_{0}$.  2:Run K-means algorithm on all rows of *U* with *K* clusters to obtain ZP, a permutation of *Z*.  3:Equation (4) gives $V\Sigma U^{\prime }ZP{\left({\left(ZP\right)}^{\prime }ZP\right)}^{-1}=\Theta P$, a permutation of Θ.

For the real case, the response matrix *R* is observed rather than the expectation response matrix $R_{0}$. We now move from the ideal scenario to the real scenario, intending to estimate *Z* and Θ when the observed response matrix *R* is a random matrix generated from an unknown distribution F satisfying Equation (2) with *K* extreme latent profiles under adLCM. The expectation of *R* is $R_{0}$ according to Equation (2) under adLCM, so intuitively, the singular values and singular vectors of *R* will be close to those of $R_{0}$. Set $\hat{R}=\hat{U}\hat{\Sigma }{\hat{V}}^{\prime }$ as the top *K* SVD of *R*, where $\hat{\Sigma }$ is a K×K diagonal matrix collecting the top *K* singular values of *R*. Write $\hat{\Sigma }=\mathrm{diag}\left(\sigma_{1}\left(R\right),\sigma_{2}\left(R\right),\ldots ,\sigma_{K}\left(R\right)\right)$. As $E\left(R\right)=R_{0}$ and the N×J matrix $R_{0}$ has *K* nonzero singular values, while the other (min(N,J)−K) singular values are zeros; we see that $\hat{R}$ should be a good approximation of $R_{0}$. Matrices $\hat{U}\in R^{N\times K},\hat{V}\in R^{J\times K}$ collect the corresponding left and right singular vectors and satisfy ${\hat{U}}^{\prime }\hat{U}={\hat{V}}^{\prime }\hat{V}=I_{K\times K}$. The above analysis implies that $\hat{U}$ should have roughly *K* distinct rows because $\hat{U}$ is a slightly perturbed version of *U*. Therefore, to obtain a good estimation of the classification matrix *Z*, we should apply the K-means algorithm on all rows of $\hat{U}$ with *K* clusters. Let $\hat{Z}$ be the estimated classification matrix returned by applying the K-means method on all rows of $\hat{U}$ with *K* clusters. Then we are able to obtain a good estimation of Θ according to Equation (4) by setting $\hat{\Theta }=\hat{V}\hat{\Sigma }{\hat{U}}^{\prime }\hat{Z}{\left({\hat{Z}}^{\prime }\hat{Z}\right)}^{-1}$. Algorithm 2, referred to as SCK, is a natural extension of the Ideal SCK from the oracle case to the real case. Note that in our SCK algorithm there are only two inputs: the observed response matrix *R* and the number of latent classes *K*, i.e., SCK does not require any tuning parameters.
**Algorithm 2** Spectral Clustering with K-means (SCK for short)**Require:** The observed response matrix $R\in R^{N\times J}$ and the number of extreme latent profiles *K*.**Ensure:** $\hat{Z}$ and $\hat{\Theta }$.  1:Obtain $\hat{R}=\hat{U}\hat{\Sigma }{\hat{V}}^{\prime }$, the top *K* SVD of *R*.  2:Run K-means algorithm on all rows of $\hat{U}$ with *K* clusters to obtain $\hat{Z}$.  3:Obtain an estimate of Θ by setting $\hat{\Theta }={\hat{R}}^{\prime }\hat{Z}{\left({\hat{Z}}^{\prime }\hat{Z}\right)}^{-1}$.

Here, we evaluate the computational cost of our SCK algorithm. The computational cost of the SVD step involved in the SCK approach is $O(\max\left(N^{2},J^{2}\right)K)$. For the K-means algorithm, its complexity is $O(NlK^{2})$ with *l* being the number of K-means iterations. In all experimental studies considered in this paper, *l* is set as 100 for the K-means algorithm. The complexity of the last step in SCK is O(JNK). Since K≪min(N,J) in this paper, as a consequence, the total time complexity of our SCK algorithm is $O(\max\left(N^{2},J^{2}\right)K)$.

## 5. Theoretical Properties

In this section, we present comprehensive theoretical properties of the SCK algorithm when the observed response matrix *R* is generated from the proposed model. Our objective is to demonstrate that the estimated classification matrix $\hat{Z}$ and the estimated item parameter matrix $\hat{\Theta }$ both concentrate around the true classification matrix *Z* and the true item parameter matrix Θ, respectively.

Let $T=\{T_{1},T_{2},\ldots ,T_{K}\}$ be the collection of true partitions for all subjects, where $T_{k}=\left\{i:Z\left(i,k\right)=1\;\mathrm{for}\;i\in \left[N\right]\right\}$ for k∈[K], i.e., $T_{k}$ is the set of the true partition of subjects into the *k*-th extreme latent profile. Similarly, let $\hat{T}=\left\{{\hat{T}}_{1},{\hat{T}}_{2},\ldots ,{\hat{T}}_{K}\right\}$ represent the collection of estimated partitions for all subjects, where ${\hat{T}}_{k}=\left\{i:\hat{Z}\left(i,k\right)=1\;\mathrm{for}\;i\in \left[N\right]\right\}$ for k∈[K]. We use the measure defined in [61] to quantify the closeness of the estimated partition $\hat{T}$ and the ground-truth partition T. Denote the *Clustering error* associated with T and $\hat{T}$ as

(7) $$\begin{array}{c} \hat{f}=\min_{\pi \in S_{K}}\max_{k\in [K]}\frac{|T_{k}\cap {\hat{T}}_{\pi (k)}^{c}\left|+\right|T_{k}^{c}\cap {\hat{T}}_{\pi (k)}|}{N_{K}}, \end{array}$$

where $S_{K}$ represents the set of all permutations of {1,2,…,K}, and ${\hat{T}}_{\pi (k)}^{c}$ and $T_{k}^{c}$ denote the complementary sets. As stated in Reference [61], $\hat{f}$ evaluates the maximum proportion of subjects in the symmetric difference of $T_{k}$ and ${\hat{T}}_{\pi (k)}$. Since the observed response matrix *R* is generated from adLCM with expectation $R_{0}$, and $\hat{f}$ measures the performance of the SCK algorithm, it is expected that SCK estimates *Z* with a small Clustering error $\hat{f}$.

For convenience, let $\rho =\max_{j\in [J],k\in [K]}\left|\Theta \left(j,k\right)\right|$ and call it the scaling parameter. Let $B=\frac{\Theta }{\rho }$, and then we have $\max_{j\in [J],k\in [K]}\left|B\left(j,k\right)\right|=1$ and $R_{0}=\rho ZB^{\prime }$. Let $\tau =\max_{i\in [N],\;j\in [J]}\left|R\left(i,j\right)-R_{0}\left(i,j\right)\right|$ and $\gamma =\max_{i\in [N],\;j\in [J]}\mathrm{Var}\left(R\left(i,j\right)\right)$ where Var(R(i,j)) means the variance of R(i,j). We require the following assumption to establish theoretical guarantees of consistency for our SCK method.

**Assumption** **1.** *Assume $\gamma \geq \frac{\tau^{2}\log\left(N+J\right)}{\max(N,J)}$.*

The following theorem presents our main result, which provides upper bounds for the error rates of our SCK algorithm under our adLCM.

**Theorem** **1.** *Under adLCM(Z,Θ,F), if Assumption 1 is satisfied, with a probability of at least $1-o({\left(N+J\right)}^{-3})$,*

$$\begin{array}{cc} \; & \hat{f}=O\left(\frac{\gamma K^{2}N_{\max}\max\left(N,J\right)\log\left(N+J\right)}{\rho^{2}N_{\min}^{2}J}\right)\;\mathrm{and}\;\frac{{∥\hat{\Theta }-\Theta P∥}_{F}}{{∥\Theta ∥}_{F}}=O\left(\frac{\sqrt{\gamma K\max(N,J)\log(N+J)}}{\rho \sqrt{N_{\min}J}}\right), \end{array}$$

*where $N_{\max}=\max_{k\in [K]}\left\{N_{k}\right\},N_{\min}=\min_{k\in [K]}\left\{N_{k}\right\}$, and P is a permutation matrix.*

Because our adLCM is distribution-free, Theorem 1 provides a general theoretical guarantee of the SCK algorithm when *R* is generated from adLCM for any distribution F as long as Equation (2) is satisfied. We can simplify Theorem 1 by considering additional conditions:

**Corollary** **1.** *Under adLCM(Z,Θ,F), when Assumption 1 holds, if we make the additional assumption that $\frac{N_{\max}}{N_{\min}}=O\left(1\right)$ and K=O(1), with a probability of at least $1-o({\left(N+J\right)}^{-3})$,*

$$\begin{array}{c} \hat{f}=O\left(\frac{\gamma \max(N,J)\log(N+J)}{\rho^{2}NJ}\right)\;\mathrm{and}\;\frac{{∥\hat{\Theta }-\Theta P∥}_{F}}{{∥\Theta ∥}_{F}}=O\left(\frac{\sqrt{\gamma \max(N,J)\log(N+J)}}{\rho \sqrt{NJ}}\right). \end{array}$$

For the case J=βN for any positive constant β, Corollary 1 implies that the SCK algorithm yields consistent estimation under adLCM since the error bounds in Corollary 1 decrease to zero as N→+∞ when ρ and distribution F are fixed.

Recall that *R* is an observed response matrix generated from a distribution F with expectation $R_{0}=Z\Theta^{\prime }=\rho ZB^{\prime }$ under adLCM and γ is the maximum variance of R(i,j) and it is closely related to the distribution F, the ranges of R,ρ,B, and γ can vary depending on the specific distribution F. The following examples provide the ranges of R,ρ,B, the upper bound of γ, and the explicit forms of error bounds in Theorem 1 for different distribution F under our adLCM. Meanwhile, based on the explicitly derived error bounds for different distribution F, we also investigate how the scaling parameter ρ influences the performance of the SCK algorithm in these examples. For all pairs (i,j) with i∈[N],j∈[J], we consider the following distributions when $E\left(R\right)=R_{0}$ in Equation (2) holds.

**Example** **1.** *Let F be a Bernoulli distributionsuch that $R\left(i,j\right)∼\mathrm{Bernoulli}(R_{0}\left(i,j\right))$, where $R_{0}\left(i,j\right)$ is the Bernoulli probability, i.e., $E\left(R\left(i,j\right)\right)=R_{0}\left(i,j\right)$. For this case, our adLCM degenerates to the LCM for data with binary responses. According to the properties of the Bernoulli distribution, we have the following conclusions.*

- *R(i,j)∈{0,1}, i.e., R(i,j) only takes two values 0 and 1.*
- *B(i,j)∈[0,1] and ρ∈(0,1] because $R_{0}\left(i,j\right)$ is a probability located in [0,1] and $\max_{i\in [N],j\in [J]}\left|B\left(i,j\right)\right|$ is assumed to be 1.*
- *τ≤1 because $\tau =\max_{i\in [N],j\in [J]}\left|R\left(i,j\right)-R_{0}\left(i,j\right)\right|\leq 1$.*
- *γ≤ρ because $\gamma =\max_{i\in [N],j\in [J]}\mathrm{Var}\left(R\left(i,j\right)\right)=\max_{i\in [N],j\in [J]}R_{0}\left(i,j\right)\left(1-R_{0}\left(i,j\right)\right)\leq \max_{i\in [N],j\in [J]}R_{0}\left(i,j\right)=\max_{i\in [N],j\in [J]}\rho \left(ZB\right)\left(i,j\right)\leq \rho$.*
- *Let τ be its upper bound 1 and γ be its upper bound ρ, Assumption 1 becomes $\rho \geq \frac{\log(N+J)}{\max(N,J)}$, which means a sparsity requirement on R because ρ controls the probability of the numbers of ones in R for this case.*
- *Let γ be its upper bound ρ in Theorem 1, then we have*
$$\begin{array}{c} \hat{f}=O\left(\frac{K^{2}N_{\max}\max\left(N,J\right)\log\left(N+J\right)}{\rho N_{\min}^{2}J}\right)\;\mathrm{and}\;\frac{{∥\hat{\Theta }-\Theta P∥}_{F}}{{∥\Theta ∥}_{F}}=O\left(\frac{\sqrt{K\max(N,J)\log(N+J)}}{\sqrt{\rho N_{\min}J}}\right). \end{array}$$
*We observe that increasing ρ leads to a decrease in SCK’s error rates when F is a Bernoulli distribution.*

**Example** **2.** *Let F be a Binomial distribution such that $R\left(i,j\right)∼\mathrm{Binomial}\left(m,\frac{R_{0}\left(i,j\right)}{m}\right)$ for any positive integer m, where R(i,j) is a random variable that reflects the number of successes in a fixed number of independent trials m with the same probability of success $\frac{R_{0}\left(i,j\right)}{m}$, i.e., $E\left(R\left(i,j\right)\right)=R_{0}\left(i,j\right)$. For this case, our adLCM reduces to the LCM for data with categorical responses. For a Binomial distribution, we have P(R(i,j)=r)=mr(R0(i,j)m)r(1−R0(i,j)m)m−r for r=0,1,2,…,m, where •• is a binomial coefficient. By the property of the Binomial distribution, we have the following conclusions.*

- *R(i,j)∈{0,1,2,…,m}.*
- *B(i,j)∈[0,1] and ρ∈(0,m] because $\frac{R_{0}\left(i,j\right)}{m}$ is a probability that has a range in [0,1].*
- *τ≤m because $\tau =\max_{i\in [N],j\in [J]}\left|R\left(i,j\right)-R_{0}\left(i,j\right)\right|\leq m$.*
- *γ≤ρ because $\gamma =\max_{i\in [N],j\in [J]}\mathrm{Var}\left(R\left(i,j\right)\right)=m\frac{R_{0}\left(i,j\right)}{m}\left(1-\frac{R_{0}\left(i,j\right)}{m}\right)=R_{0}\left(i,j\right)\left(1-\frac{R_{0}\left(i,j\right)}{m}\right)\leq \rho$.*
- *Let τ be its upper bound m and γ be its upper bound ρ, then Assumption 1 becomes $\rho \geq \frac{m^{2}\log\left(N+J\right)}{\max(N,J)}$ which provides a lower bound requirement of the scaling parameter ρ.*
- *Let γ be its upper bound ρ in Theorem 1, then we obtain the exact forms of error bounds for SCK when F is a Binomial distribution, and we observe that increasing ρ reduces SCK’s error rates.*

**Remark** **5.** *When F is the Binomial distribution and Condition 1 of [28] holds (K=O(1), J=O(N), $N_{\max}/N_{\min}=O\left(1\right)$), the theoretical upper bounds for $\hat{f}$ and $\frac{\left|\hat{\Theta }-\Theta P\right|F}{|\Theta |F}$ correspond to the squared error bounds established in Theorem 1 of [28]. This optimality guarantee is consistent with Assumption 1, which aligns with Assumption 1 in [28]. The relationship arises because the theoretical results in [28] apply to the overlapping case (each subject can belong to multiple latent classes). Under mild conditions, spectral clustering methods in the non-overlapping case (each subject belongs to only one latent class) typically achieve an error bound that is the square of the bound for spectral methods in the overlapping case, while requiring similar sparsity levels. This phenomenon is generally observed in the area of community detection: for instance, spectral methods for non-overlapping networks [67,68,69,70] exhibit squared error bounds relative to those for overlapping networks [64,65,71,72], despite comparable sparsity requirements. We refer readers to [73] for further discussion of this phenomenon.*

**Example** **3.** *Let F be a Poisson distribution such that $R\left(i,j\right)∼\mathrm{Poisson}(R_{0}\left(i,j\right))$, where $R_{0}\left(i,j\right)$ is the Poisson parameter, i.e., $E\left(R\left(i,j\right)\right)=R_{0}\left(i,j\right)$. By the properties of the Poisson distribution, the following conclusions can be obtained.*

- *R(i,j)∈N, i.e., R(i,j) is an nonnegative integer.*
- *B(i,j)]∈[0,1] and ρ∈(0,+∞) because Poisson distribution can take any positive value for its mean.*
- *τ is an unknown positive value because we cannot know the exact upper bound of R(i,j) when R is obtained from the Poisson distribution under the adLCM.*
- *γ≤ρ because $\gamma =\max_{i\in [N],j\in [J]}\mathrm{Var}\left(R\left(i,j\right)\right)=\max_{i\in [N],j\in [J]}R_{0}\left(i,j\right)\leq \rho$.*
- *Let γ be its upper bound ρ, then Assumption 1 becomes $\rho \geq \frac{\tau^{2}\log\left(N+j\right)}{\max(N,J)}$ which is a lower bound requirement of ρ.*
- *Let γ be its upper bound ρ in Theorem 1 which obtains the exact forms of error bounds for the SCK algorithm when F is a Poisson distribution. It is easy to observe that increasing ρ leads to a decrease in SCK’s error rates.*

**Example** **4.** *Let F be a Normal distribution such that $R\left(i,j\right)∼\mathrm{Normal}(R_{0}\left(i,j\right),\sigma^{2})$, where $R_{0}\left(i,j\right)$ is the mean ( i.e., $E\left(R\left(i,j\right)\right)=R_{0}\left(i,j\right)$) and $\sigma^{2}$ is the variance parameter for Normal distribution. For this case, we have*

- *R(i,j)∈R, i.e., R(i,j) is a real value.*
- *B(i,j)∈[−1,1] and ρ∈(0,+∞) because the mean of Normal distribution can take any value. Note that, unlike the cases when F is Bernoulli or Poisson, B can have negative elements for the Normal distribution case.*
- *Similar to Example 3, τ is an unknown positive value.*
- *$\gamma =\sigma^{2}$ because $\gamma =\max_{i\in [N],j\in [J]}\mathrm{Var}\left(R\left(i,j\right)\right)=\max_{i\in [N],j\in [J]}\sigma^{2}=\sigma^{2}$ for Normal distribution.*
- *Let γ be its exact value $\sigma^{2}$, then Assumption 1 becomes $\sigma^{2}\max\left(N,J\right)\geq \tau^{2}\log\left(N+J\right)$ which means that max(N,J) should be set larger than $\frac{\tau^{2}\log\left(N+J\right)}{\sigma^{2}}$ for our theoretical analysis.*
- *Let γ be its exact value $\sigma^{2}$ in Theorem 1 which provides the exact forms of error bounds for SCK. We observe that increasing the scaling parameter ρ (or decreasing the variance $\sigma^{2}$) reduces SCK’s error rates.*

**Example** **5.** *Let F be an Exponential distribution such that $R\left(i,j\right)∼\mathrm{Exponential}\left(\frac{1}{R_{0}\left(i,j\right)}\right)$, where $\frac{1}{R_{0}\left(i,j\right)}$ is the Exponential parameter, i.e., $E\left(R\left(i,j\right)\right)=R_{0}\left(i,j\right)$. For this case, we have*

- *$R\left(i,j\right)\in R_{+}$, i.e., R(i,j) is a positive value.*
- *B(i,j)∈(0,1] and ρ∈(0,+∞) because the mean of Exponential distribution can be any positive value.*
- *Similar to Example 3, τ is an unknown positive value.*
- *$\gamma \leq \rho^{2}$ because $\gamma =\max_{i\in [N],j\in [J]}\mathrm{Var}\left(R\left(i,j\right)\right)=\max_{i\in [N],j\in [J]}R_{0}^{2}\left(i,j\right)\leq \rho^{2}$ for Exponential distribution.*
- *Let γ be its upper bound $\rho^{2}$, then Assumption 1 becomes $\rho^{2}\geq \tau^{2}\log\left(N+J\right)/\max\left(N,J\right)$, a lower bound requirement of ρ.*
- *Let γ be its upper bound $\rho^{2}$ in Theorem 1; the theoretical bounds demonstrate that ρ vanishes, which indicates that increasing ρ has no significant impact on the error rates of SCK.*

**Example** **6.** *Let F be a Uniform distribution such that $R\left(i,j\right)∼\mathrm{Uniform}(0,2R_{0}\left(i,j\right))$, where $E\left(R\left(i,j\right)\right)=\frac{0+2R_{0}\left(i,j\right)}{2}=R_{0}\left(i,j\right)$ holds immediately. For this case, we have*

- *R(i,j)∈(0,2ρ) because $2R_{0}\left(i,j\right)\leq 2\rho$.*
- *B(i,j)∈(0,1] and ρ∈(0,+∞) because $\mathrm{Uniform}(0,2R_{0}\left(i,j\right))$ allows $2R_{0}\left(i,j\right)$ to be any positive value.*
- *τ is an unknown positive value with an upper bound 2ρ.*
- *$\gamma \leq \frac{\rho^{2}}{3}$ because $\gamma =\max_{i\in [N],j\in [J]}\mathrm{Var}\left(R\left(i,j\right)\right)=\max_{i\in [N],j\in [J]}\frac{{\left(2R_{0}\left(i,j\right)-0\right)}^{2}}{12}=$$\max_{i\in [N],j\in [J]}\frac{R_{0}^{2}\left(i,j\right)}{3}\leq \frac{\rho^{2}}{3}$ for Uniform distribution.*
- *Let γ be its upper bound $\frac{\rho^{2}}{3}$, then Assumption 1 becomes $\rho^{2}\geq 3\tau^{2}\log\left(N+J\right)/\max\left(N,J\right)$, a lower bound requirement of ρ.*
- *Since ρ disappears in the error bounds when we let $\gamma =\frac{\rho^{2}}{3}$ in Theorem 1, increasing ρ does not significantly influence SCK’s error rates, a conclusion similar to Example 5.*

**Example** **7.** *Our adLCM can also model a signed response matrix by setting $P\left(R\left(i,j\right)=1\right)=\frac{1+R_{0}\left(i,j\right)}{2}$ and $P\left(R\left(i,j\right)=-1\right)=\frac{1-R_{0}\left(i,j\right)}{2}$, where $E\left(R\left(i,j\right)\right)=\frac{1+R_{0}\left(i,j\right)}{2}-\frac{1-R_{0}\left(i,j\right)}{2}=R_{0}\left(i,j\right)$ and Equation (2) holds surely. For the signed response matrix, we have*

- *R(i,j)∈{−1,1}, i.e., R(i,j) only takes two values −1 and 1.*
- *B(i,j)∈[−1,1] and ρ∈(0,1] because $\frac{1+R_{0}\left(i,j\right)}{2}$ and $\frac{1-R_{0}\left(i,j\right)}{2}$ are two probabilities which should be in the range [0,1]. Note that, similar to Example 4, B(i,j) can be negative for the signed response matrix.*
- *τ≤2 because R(i,j)∈{−1,1} and $R_{0}\left(i,j\right)\in \left[-1,1\right]$.*
- *γ≤1 because $\gamma =\max_{i\in [N],j\in [J]}\mathrm{Var}\left(R\left(i,j\right)\right)=\max_{i\in [N],j\in [J]}\left(1-R_{0}^{2}\left(i,j\right)\right)\leq 1$.*
- *When setting τ=2 and γ=1, Assumption 1 turns to be max(N,J)≥4log(N+J).*
- *Setting γ as its upper bound 1 in Theorem 1 gives that increasing ρ reduces SCK’s error rates.*

## 6. Simulation Studies

In this section, we conduct extensive simulation experiments to evaluate the effectiveness of the proposed method and validate our theoretical results in Examples 1–7.

### 6.1. Baseline Method

More than the SCK algorithm, here we briefly provide an alternative spectral method that can also be applied to fit our adLCM. Recall that $R_{0}=Z\Theta^{\prime }$ under adLCM; it is easy to see that $R_{0}\left(i,:\right)=R_{0}\left(\bar{i},:\right)$ when two distinct subjects *i* and $\bar{i}$ belong to the same extreme latent profile for $i,\bar{i}\in \left[N\right]$. Therefore, the population response matrix $R_{0}$ features K disparate rows, and running the K-means approach on all rows of $R_{0}$ with *K* clusters can faithfully recover the classification matrix *Z* in terms of a permutation of the *K* extreme latent profiles. $R_{0}=Z\Theta^{\prime }$ also gives that $\Theta =R_{0}^{\prime }Z{\left(Z^{\prime }Z\right)}^{-1}$, which suggests the following ideal algorithm called Ideal RMK Algorithm 3).
**Algorithm 3** Ideal RMK**Require:** $R_{0},K$.**Ensure:** A permutation of *Z* and Θ.  1:Run K-means algorithm on all rows of $R_{0}$ with *K* clusters to obtain ZP, a permutation of *Z*.  2:Compute $R_{0}^{\prime }ZP{\left({\left(ZP\right)}^{\prime }ZP\right)}^{-1}=\Theta P$, a permutation of Θ.

Algorithm 4, called RMK, is a natural generalization of the Ideal RMK from the oracle case to the real case because $E\left(R\right)=R_{0}$ under adLCM. Unlike the SCK method, the RMK method does not need to obtain the SVD of the observed response matrix *R*.
**Algorithm 4** Response Matrix with K-means (RMK for short)**Require:** R,K.**Ensure:** $\hat{Z},\hat{\Theta }$.  1:Run K-means algorithm on all rows of *R* with *K* clusters to obtain $\hat{Z}$.  2:Obtain an estimate of Θ by setting $\hat{\Theta }=R^{\prime }\hat{Z}{\left({\hat{Z}}^{\prime }\hat{Z}\right)}^{-1}$.

The computational cost of the first step in RMK is O(lNJK), where *l* denotes the number of iterations for the K-means algorithm. The complexity of the second step in RMK is O(JNK). Therefore, the overall computational cost of RMK is O(lNJK). When J=βN for a constant value β∈(0,1], the complexity of RMK is $O(\beta lKN^{2})$, and it is larger than the SCK’s complexity $O(KN^{2})$ when βl>1. Therefore, SCK runs faster than RMK when βl>1, as confirmed by our numerical results in this section.

We also compare our SCK and RMK approaches with several existing methods to highlight their superior performance. The comparative methods include the probabilistic latent component analysis (PLCA) algorithm [20] (an expectation maximization method), the nonnegative matrix factorization (NMF) technique [24], and the HeteroClustering (HC) algorithm [29] (a spectral clustering approach).

### 6.2. Evaluation Metric

For the classification of subjects, when the true classification matrix *Z* is known, to evaluate how good the quality of the partition of the subjects into extreme latent profiles is, four metrics are considered including the Clustering error $\hat{f}$ computed by Equation (7). The other three popular evaluation criteria are Hamming error [74], normalized mutual information (NMI) [75,76,77,78], and adjusted rand index (ARI) [78,79,80].

- Hamming error is defined as
  $$\begin{array}{c} \mathrm{Hamming}\;\mathrm{error}=N^{-1}\min_{P\in P_{K}}{∥\hat{Z}-ZP∥}_{0}, \end{array}$$
  where $P_{K}$ denotes the collection of all *K*-by-*K* permutation matrices. Hamming error falls within the range [0,1], and a smaller Hamming error indicates better classification performance.
- Let *C* be a K×K confusion matrix such that C(k,l) is the number of common subjects between $T_{k}$ and ${\hat{T}}_{l}$ for k,l∈[K]. NMI is defined as
  $$\begin{array}{c} \mathrm{NMI}=\frac{-2\sum_{k,l}C\left(k,l\right)\log\left(\frac{C(k,l)N}{C_{k.}C_{.l}}\right)}{\sum_{k}C_{k.}\log\left(\frac{C_{k.}}{N}\right)+\sum_{l}C_{.l}\log\left(\frac{C_{.l}}{N}\right)}, \end{array}$$
  where $C_{k.}=\sum_{m=1}^{K}C\left(k,m\right)$ and $C_{.l}=\sum_{m=1}^{K}C\left(m,l\right)$. NMI is in the range [0,1] and the larger it is, the better it is.
- ARI is defined as
  ARI=∑k,lC(k,l)2−∑kCk.2∑lC.l2N212[∑kCk.2+∑lC.l2]−∑kCk.2∑lC.l2N2,
  where .. is a binomial coefficient. ARI falls within the range [−1,1] and the larger it is, the better it is.

For the estimation of Θ, we use the Relative $l_{1}$ error and the Relative $l_{2}$ error to evaluate the performance. The two criteria are defined as

$$\begin{array}{c} \mathrm{Relative}\;l_{1}\;\mathrm{error}=\min_{P\in P_{K}}\frac{{∥\hat{\Theta }-\Theta P∥}_{1}}{{∥\Theta ∥}_{1}}\;\mathrm{and}\;\mathrm{Relative}\;l_{2}\;\mathrm{error}=\min_{P\in P_{K}}\frac{{∥\hat{\Theta }-\Theta P∥}_{F}}{{∥\Theta ∥}_{F}}. \end{array}$$

The smaller both values are the better.

### 6.3. Synthetic Data

We conduct numerical studies to examine the accuracy and the efficiency of the aforementioned approaches by changing the scaling parameter ρ and the number of subjects *N*. Unless specified, in all computer-generated response matrices, we set $K=3,J=\frac{N}{5}$, and the N×K classification matrix *Z* is generated such that each subject belongs to one of the *K* extreme latent profiles with equal probability. For distributions that require *B*’s entries to be nonnegative, we let B(j,k)=rand(1) for j∈[J],k∈[K], where rand(1) is a random value simulated from the uniform distribution on [0,1]. For a Normal distribution and signed response matrix that allow *B* to have negative entries, we let B(j,k)=2rand(1)−1 for j∈[J],k∈[K], i.e., B(j,k) is in the range [−1,1]. Set $B_{\max}=\max_{j\in [J],k\in [K]}\left|B\left(j,k\right)\right|$ because the generation process of *B* makes |B(j,k)|∈[0,1], but it cannot guarantee that $B_{\max}=1$, which is required in the definition of *B*. Therefore, we update *B* by $\frac{B}{B_{\max}}$. For the scaling parameter ρ and the number of subjects *N*, they are set independently for each distribution. After setting all model parameters (K,N,J,Z,B,ρ), we can generate the observed response matrix *R* from distribution F with expectation $R_{0}=Z\Theta^{\prime }=\rho ZB^{\prime }$ under our adLCM. By applying each method to *R* with *K* extreme latent profiles, we can compute the evaluation metrics of each method. In every simulation scenario, we generate 50 independent replicates and report the mean of Clustering error (as well as Hamming error, NMI, ARI, Relative $l_{1}$ error, Relative $l_{2}$ error, and running time) computed from the 50 repetitions for each method. All numerical results in this paper are reported using MATLAB R2024b on a standard personal computer (Thinkpad X1 Carbon Gen 8).

#### 6.3.1. Bernoulli Distribution

When $R\left(i,j\right)∼\mathrm{Bernoulli}(R_{0}\left(i,j\right))$ for i∈[N], j∈[J], we consider the following two simulations.

Simulation 1(a): changing ρ. Set N=500. For the Bernoulli distribution, the scaling parameter ρ should be set within the range (0,1] according to Example 1. Here, for simulation studies, we let ρ be in the range {0.1,0.2,0.3,…,1}.

Simulation 1(b): changing N. Let ρ=0.1 and N be in the range {1000,2000,…,5000}.

**Figure 1:**
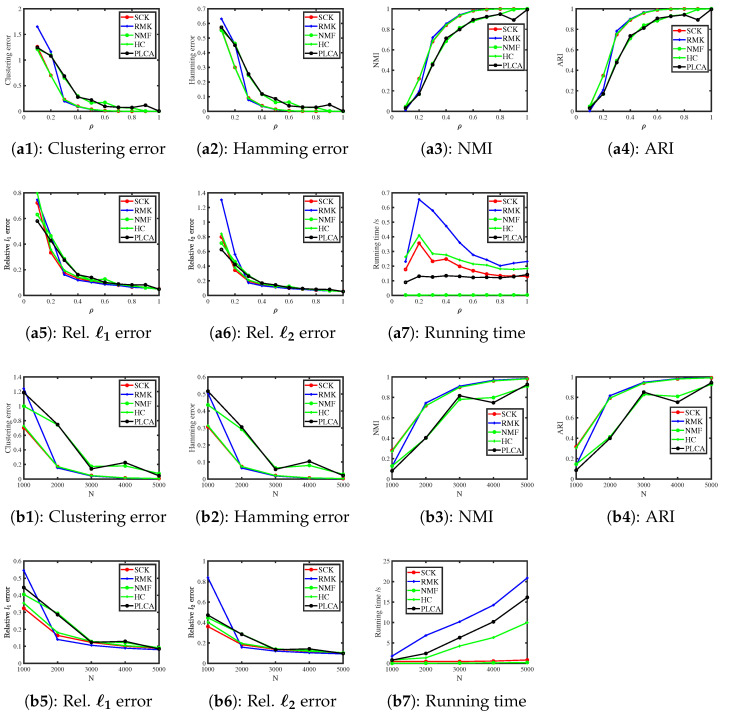
Numerical results of Simulation 1.

The results are presented in Figure 1. We observe that SCK and HC outperform the other three methods, while NMF and PLCA perform the poorest in estimating (Z,Θ). As for running time, SCK and NMF run faster than their competitors across all settings. All methods achieve better performances as ρ increases, which conforms to our analysis in Example 1. Additionally, all algorithms enjoy better performances when the number of subjects N increases, as predicted by our analysis following Corollary 1.

#### 6.3.2. Binomial Distribution

When $R\left(i,j\right)∼\mathrm{Binomial}\left(m,\frac{R_{0}\left(i,j\right)}{m}\right)$ for i∈[N],j∈[J], we consider the following two simulations.

Simulation 2(a): changing ρ. Set N=500 and m=5. Recall that ρ’s range is (0,m] when F is a Binomial distribution according to Example 2; here, we let ρ be in the range {0.2,0.4,0.6,…,2}.

Simulation 2(b): changing N. Let ρ=0.1,m=5, and N be in the range {1000,2000,…,5000}.

**Figure 2:**
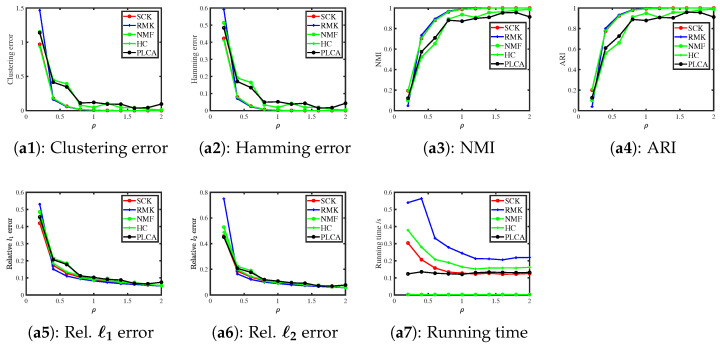

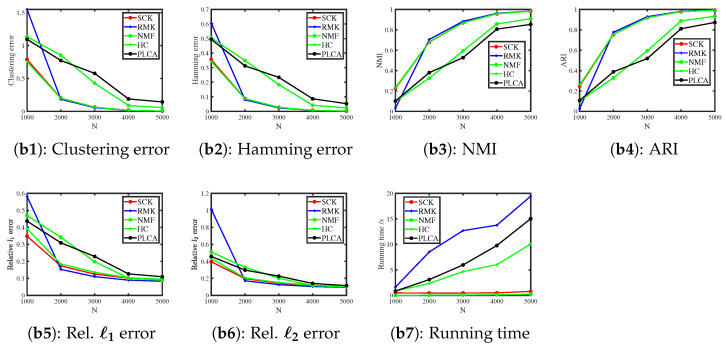
Numerical results of Simulation 2.

Figure 2 presents the corresponding results. We note that SCK, RMK, and HC enjoy similar error rates and they outperform NMF and PLCA in estimating (Z,Θ). NMF runs slightly faster than SCK, while SCK runs faster than the other three approaches for this simulation. Meanwhile, increasing ρ (and N) decreases error rates for all methods, which confirms our findings in Example 2 and Corollary 1.

#### 6.3.3. Poisson Distribution

When $R\left(i,j\right)∼\mathrm{Poisson}(R_{0}\left(i,j\right))$ for i∈[N],j∈[J], we consider the following two simulations.

Simulation 3(a): changing ρ. Set N=500. Example 3 says that the theoretical range of ρ is (0,+∞) when F is a Poisson distribution. Here, we let ρ be in the range {0.2,0.4,0.6,…,2}.

Simulation 3(b): changing N. Let ρ=0.1 and N be in the range {1000,2000,…,5000}.

**Figure 3:**
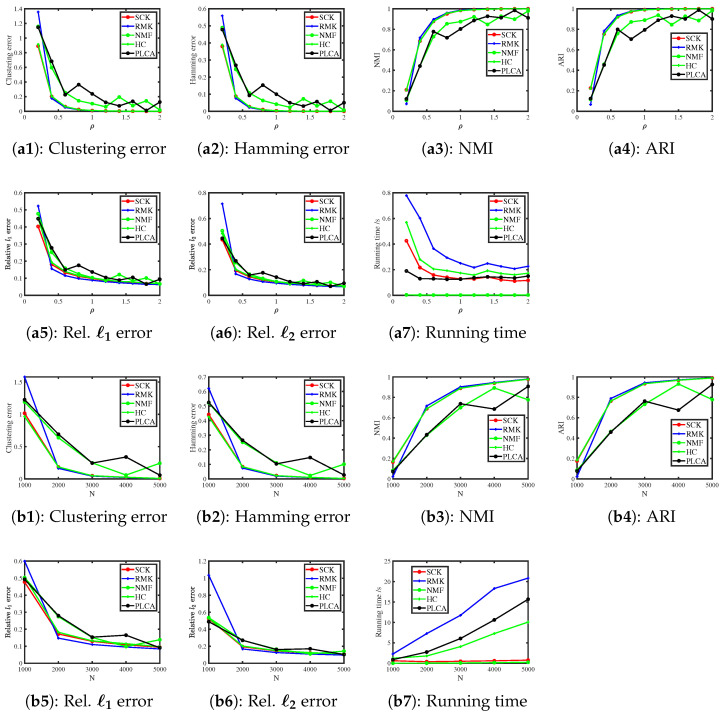
Numerical results of Simulation 3.

Figure 3 displays the numerical results of Simulation 3(a) and Simulation 3(b). The results are similar to those of the Binomial distribution case: SCK, RMK, and HC perform similarly, while NMF and PLCA perform poorer in estimating (Z,Θ). SCK runs slightly slower than NMF and both methods run faster than their competitors. All methods perform better as ρ and N increase, which supports our analysis in Example 3 and Corollary 1.

#### 6.3.4. Normal Distribution

When $R\left(i,j\right)∼\mathrm{Normal}(R_{0}\left(i,j\right),\sigma^{2})$ for i∈[N],j∈[J], we consider the following two simulations.

Simulation 4(a): changing ρ. Set N=500 and $\sigma^{2}=2$. According to Example 4, the scaling parameter ρ can be set as any positive value when F is a Normal distribution. Here, we let ρ be in the range {0.2,0.4,0.6,…,2}.

Simulation 4(b): changing N. Let $\rho =0.5,\sigma^{2}=2$, and N be in the range {1000,2000,…,5000}.

**Figure 4:**
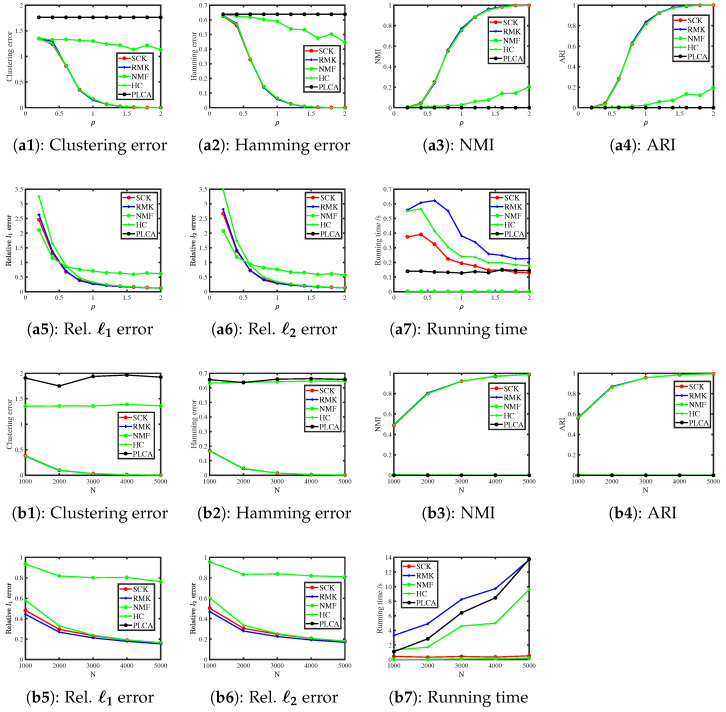
Numerical results of Simulation 4.

Figure 4 shows the results. We see that SCK, RMK, and HC have similar performances in estimating model parameters (Z,Θ), while NMF and PLCA fail to estimate parameters in this simulation. For running time, SCK runs faster than RMK and HC. Additionally, the error rates of SCK, RMK, and HC decrease when the scaling parameter ρ and the number of subjects N increase, supporting our findings in Example 4 and Corollary 1.

#### 6.3.5. Exponential Distribution

When $R\left(i,j\right)∼\mathrm{Exponential}\left(\frac{1}{R_{0}\left(i,j\right)}\right)$ for i∈[N],j∈[J], we consider the following two simulations.

Simulation 5(a): changing ρ. Set N=300. According to Example 5, the range of the scaling parameter ρ is (0,+∞) when F is an Exponential distribution. Here, we let ρ be in the range {1,2,…,20} for our numerical studies.

Simulation 5(b): changing N. Let ρ=1 and N be in the range {300,600,…,3000}.

**Figure 5:**
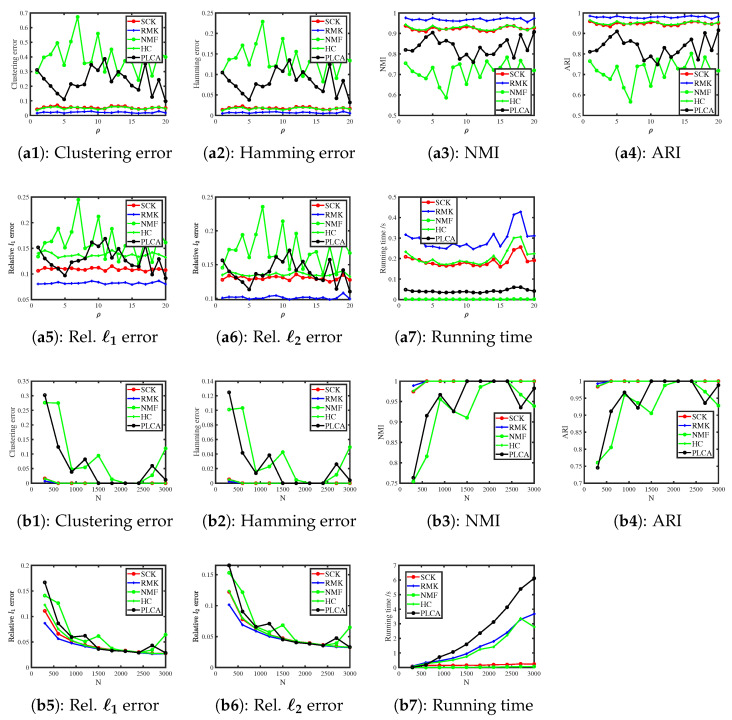
Numerical results of Simulation 5.

Figure 5 displays the results. We see that SCK, RMK, and HC provide satisfactory estimations for Z and Θ for their small error rates, large NMI, and large ARI, while NMF and PLCA perform poorer. For running time, NMF and SCK run faster than their competitors for large N. Meanwhile, we find that increasing ρ does not significantly influence the performances of these methods and this verifies our theoretical analysis in Example 5 that ρ disappears in the theoretical upper bounds of error rates by setting $\gamma =\rho^{2}$ in Theorem 1 for Exponential distribution. Furthermore, when we increase N, all methods perform better and this supports our analysis after Corollary 1.

#### 6.3.6. Uniform Distribution

When $R\left(i,j\right)∼\mathrm{Uniform}(0,2R_{0}\left(i,j\right))$ for i∈[N],j∈[J], we consider the following two simulations.

Simulation 6(a): changing ρ. Set N=120. According to Example 6, the scaling parameter ρ can be set as any positive value when F is a Uniform distribution. Here, we let ρ be in the range {1,2,…,20}.

Simulation 6(b): changing N. Let ρ=1 and N be in the range {300,600,…,3000}.

**Figure 6:**
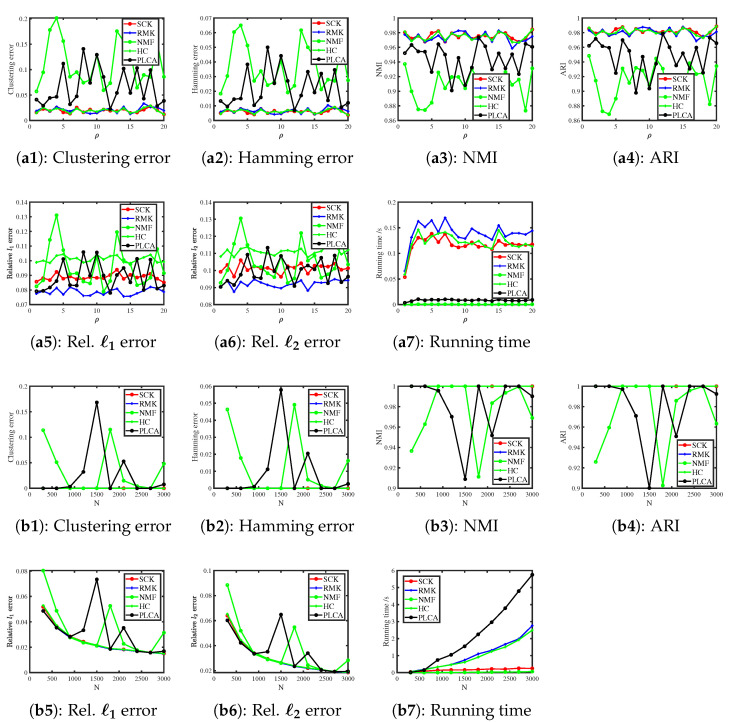
Numerical results of Simulation 6.

Figure 6 displays the numerical results. We see that increasing ρ does not significantly decrease or increase the estimation accuracies of these methods, which verifies our theoretical analysis in Example 6. For all settings, SCK runs faster than RMK and HC. When increasing N, the Clustering error and Hamming error (NMI and ARI) for SCK, RMK, and HC are 0 (1), and this suggests that they return the exact estimation of the classification matrix Z. This phenomenon occurs because N is set quite large for Uniform distribution in Simulation 6(b). For the estimation of Θ, the error rates for SCK, RMK, and HC decrease when we increase N and this is consistent with our findings following Corollary 1. The numerical results for running time are similar to previous simulations, and we omit the detailed analysis here for brevity.

#### 6.3.7. Signed Response Matrix

For signed response matrices when $P\left(R\left(i,j\right)=1\right)=\frac{1+R_{0}\left(i,j\right)}{2}$ and $P\left(R\left(i,j\right)=-1\right)=\frac{1-R_{0}\left(i,j\right)}{2}$ for i∈[N],j∈[J], we consider the following two simulations.

Simulation 7(a): changing ρ. Set N=500. Recall that the theoretical range of the scaling parameter ρ is (0,1] for signed response matrices according to our analysis in Example 7; here, we let ρ be in the range {0.1,0.2,…,1}.

Simulation 7(b): changing N. Let ρ=0.2 and N be in the range {1000,2000,…,5000}.

**Figure 7:**
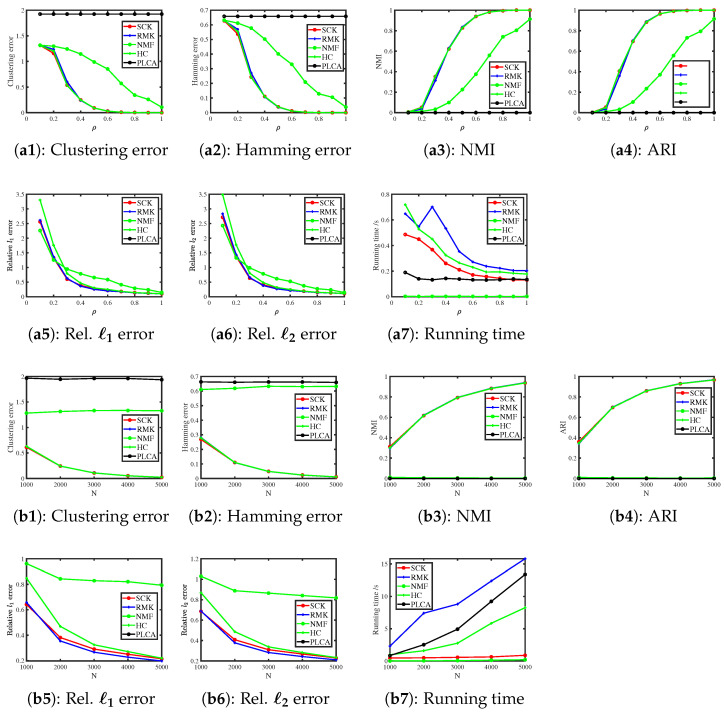
Numerical results of Simulation 7.

Figure 7 shows the results. We see that increasing ρ and N improves the estimation accuracies of SCK, HC, and RMK, which confirms our analysis in Example 7 and Corollary 1. Meanwhile, PLCA and NMF almost fail to estimate Z in this simulation. Additionally, it is easy to see that SCK, RMK, and HC enjoy similar performances in estimating Z and Θ, and SCK requires less computation time compared to RMK and HC.

#### 6.3.8. Simulated Arbitrary-Distribution Response Matrices

For visuality, we plot two response matrices R generated from the Normal distribution and the Poisson distribution under adLCM. Let $K=2,N=16,J\;=\;10,\sigma^{2}\;=\;1,\ell \left(i\right)\;=\;1,$ℓ(i+8)=2 for i∈[8], and Θ(j,1)=100,Θ(j,2)=110−10j for j∈[10]. Because $R_{0}=Z\Theta^{\prime }$ has been set, we can generate R under different distributions with expectation $R_{0}$ under the proposed adLCM. Here, we consider the following two settings.

Simulation 8 (a): When $R\left(i,j\right)∼\mathrm{Normal}(R_{0}\left(i,j\right),\sigma^{2})$ for i∈[N],j∈[J].

Simulation 8 (b): When $R\left(i,j\right)∼\mathrm{Poisson}(R_{0}\left(i,j\right))$ for i∈[N],j∈[J].

**Figure 8:**
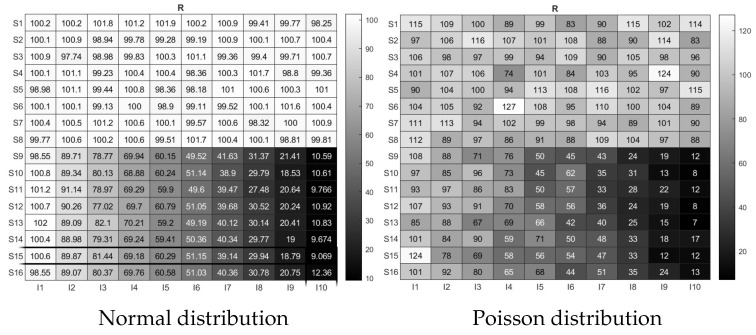
Illustration for response matrices R generated from adLCM. In both panels, Si denotes subject i and Ij denotes item j for i∈[16],j∈[10].

**Figure 9:**
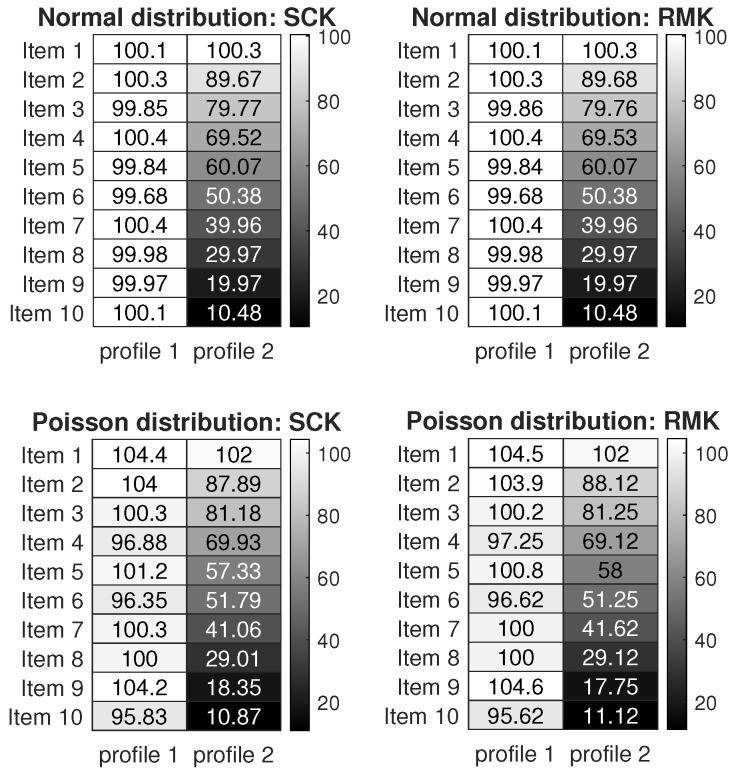
Heatmap of the estimated item parameter matrix $\hat{\Theta }$ of SCK and RMK for R in Figure 8.

Figure 8 displays the response matrices R generated for Simulation 8 (a) and 8 (b), respectively. Error rates of these methods for the corresponding observed response matrices are displayed in Table 1. We also plot the estimated item matrix $\hat{\Theta }$ for our SCK and RMK in Figure 9. We see that all approaches exactly recover Z from R, while they estimate Θ with slight perturbations. Meanwhile, since Z,Θ, and K are known for this simulation, the R provided in Figure 8 can be regarded as benchmark response matrices, and readers can apply SCK and RMK (and other methods) to R to check their effectiveness in estimating Z and Θ.

In summary, we conduct extensive numerical experiments across a wide spectrum of data-generating distributions to evaluate the SCK and RMK methods compared against various benchmarks. The proposed SCK algorithm consistently demonstrates superior performance and efficiency for latent class analysis of various types of response. Across all simulation settings, SCK achieves highly accurate estimates of both the latent class memberships Z and item parameters Θ, often matching or exceeding the performance of alternative methods like RMK and HC while significantly outperforming NMF and PLCA. Notably, SCK demonstrates exceptional computational efficiency, consistently running faster than RMK and HC, and substantially faster than the EM-based PLCA method, which is the slowest across all scenarios. Furthermore, the experiments reveal a critical limitation of NMF and PLCA: the two methods consistently fail to provide reliable parameter estimates when the response matrix contains negative values, as evidenced by their poor performance in simulations involving Normal distributions and signed response matrices. In contrast, SCK robustly handles all distributions, including those with negative or continuous responses, confirming its versatility as a powerful and efficient tool for the analysis of data under the proposed adLCM framework.

## 7. Real Data Applications

As the main goal of this paper is to introduce the proposed adLCM and the SCK algorithm for arbitrary-distribution response matrices, this section reports empirical results on two datasets. Because the true classification matrix and the true item parameter matrix are unknown for real data, and SCK runs much faster than RMK and HC though they may perform similarly in simulations, we only report the outcomes of the SCK approach. For real-world datasets, the number of extreme latent profiles K is often unknown. Here, we infer K for real-world data using the following strategy:

(8) $$\begin{array}{c} K=\arg\;\min_{k\in [\mathrm{rank}(R)]}∥R-\hat{Z}{\hat{\Theta }}^{\prime }∥, \end{array}$$

where $\hat{Z}$ and $\hat{\Theta }$ are outputs in Algorithm 2 with inputs R and k. The method specified in Equation (8) selects K by picking the one that minimizes the spectral norm difference between R and $\hat{Z}{\hat{\Theta }}^{\prime }$. The determination of the number of extreme latent profiles K in our adLCM in a rigorous manner with theoretical guarantees remains a future direction.

### 7.1. International Personality Item Pool (IPIP) Personality Test Data

**Figure 10:**
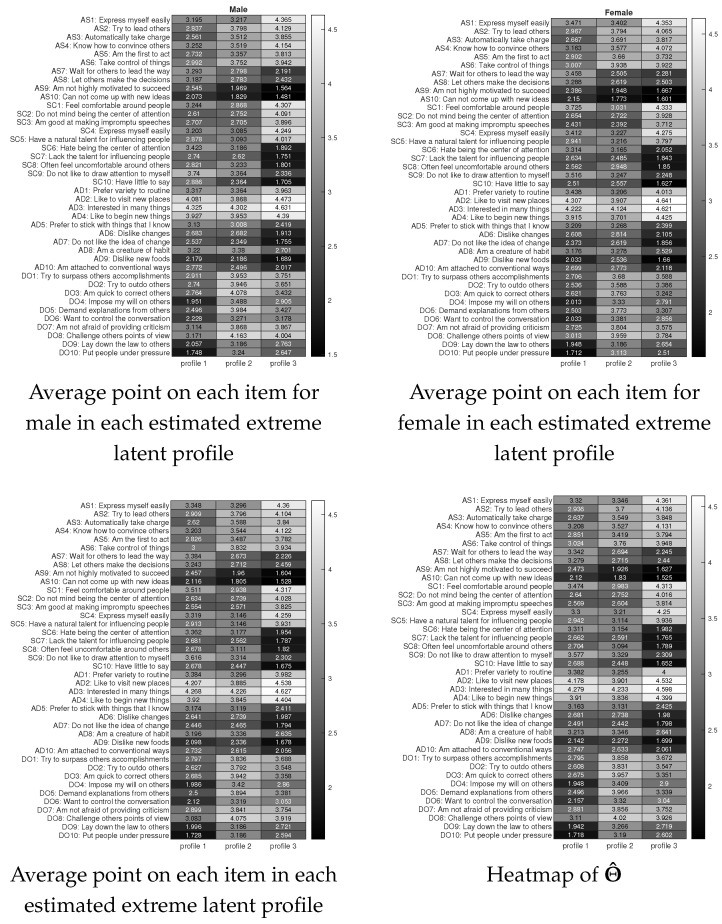
Numerical results for the IPIP data.

**Background.** We apply SCK to an experiment personality test dataset called the International Personality Item Pool (IPIP) personality test, which is obtainable for download at https://openpsychometrics.org/_rawdata/ (accessed on 11 August 2025). This data consists of 1005 subjects and 40 items. The IPIP data also records the age and gender of each subject. After dropping subjects with missing entries in their responses, age, or gender, and dropping two subjects that are neither male nor female, there are 896 subjects left, i.e., N=896,J=40. All items are rated on a 5-point scale, where 1 = Strongly disagree, 2 = Disagree, 3 = Neither agree not disagree, 4 = Agree, 5 = Strongly agree, i.e., $R\in {\left\{1,2,3,4,5\right\}}^{896\times 40}$, a response matrix. Items 1–10 measure the personality factor Assertiveness (short as “AS”); Items 11–20 measure the personality factor Social confidence (short as “SC”); Items 21–30 measure the personality factor Adventurousness (short as “AD”); and Items 31–40 measure the personality factor Dominance (short as “DO”). The details of each item are depicted in Figure 10.

**Analysis.** We apply Equation (8) to infer K for the IPIP dataset and find that the estimated value of K is 3. We then apply the SCK algorithm to the response matrix R with K=3 to obtain the 896×3 matrix $\hat{Z}$ and the 40×3 matrix $\hat{\Theta }$. The running time for SCK on this dataset is around 0.2 s.

**Results.** For convenience, we denote the estimated three extreme latent profiles as profile 1, profile 2, and profile 3. Based on $\hat{Z}$ and the information of age and gender, we can obtain some basic information (shown in Table 2) such as the size of each profile, number of males (females) in each profile, and the average age of males (and females) in each profile. From Table 2, we see that the number of females is larger than that of males for profile 1, while profiles 2 and 3 have more males. The average age of males (and females) in profile 2 is smaller than that of profiles 1 and 3, while the average age of females in profile 3 is the largest. We can also obtain the average point on each item for males (and females) in each estimated extreme latent profile and the results are shown in Figure 10. We observe that males in profile 3 tend to be more confident, more creative, more social, and more open to changes than males in profiles 1 and 2; males in profile 3 are more (less) dominant than males in profile 1 (profile 2). Males in profile 2 are more confident, creative, social, open to changes, and dominant than males in profile 1. Meanwhile, in the three estimated extreme latent profiles, females enjoy similar personalities to males. We also find that males in profile 3 (profile 2) are more (less) confident, creative, social, open to changes, and dominant than females in profile 3 (profile 2). Furthermore, it is interesting to see that, though males in profile 1 are less confident, creative, social, and open to changes than females in profile 1, they are more dominant than females in profile 1. We also plot the average point on each item in each estimated extreme latent profile regardless of gender in Figure 10 where we can draw similar conclusions as those for males. In Figure 10, we also plot the heatmap of the estimated item parameter matrix $\hat{\Theta }$. By comparing these results shown in Figure, we see that the (j,k)-th element in the matrix shown in the third panel of Figure 10 is close to $\hat{\Theta }\left(j,k\right)$ for j∈[40],k∈[3]. Such a result implies that the behavior differences on each item for every extreme latent profile are governed by the item parameter matrix Θ.

**Remark** **6.** *Recall that $E\left(R\right)=R_{0}=Z\Theta^{\prime }$ under the adLCM; then we have $R_{0}\left(i,j\right)=\Theta \left(j,\ell \left(i\right)\right)$ for i∈[N],j∈[J]. Then we have $\sum_{\ell (i)\equiv k}R_{0}\left(i,j\right)=\sum_{\ell (i)\equiv k}\Theta \left(j,\ell \left(i\right)\right)=\sum_{\ell (i)\equiv k}\Theta \left(j,k\right)=N_{k}\Theta \left(j,k\right)$, which gives that $\Theta \left(j,k\right)=\frac{\sum_{\ell (i)\equiv k}R_{0}\left(i,j\right)}{N_{k}}$ for k∈[K]. This explains why the average value on the j-th item in the k-th estimated extreme latent profile approximates $\hat{\Theta }\left(j,k\right)$ for j∈[J],k∈[K].*

### 7.2. Big Five Personality Test with Random Number (BFPTRN) Data

**Figure 11:**
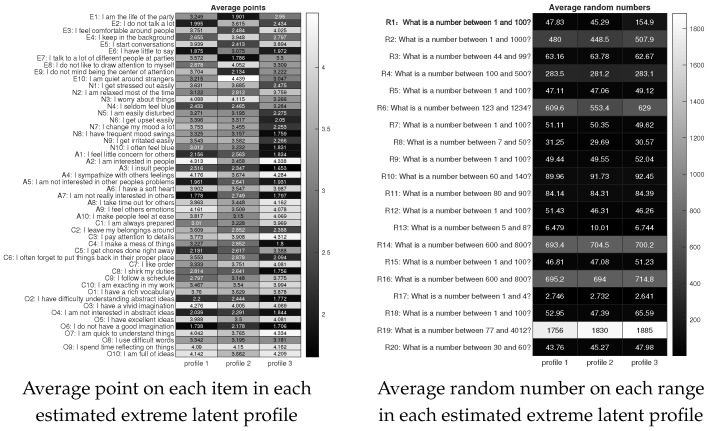

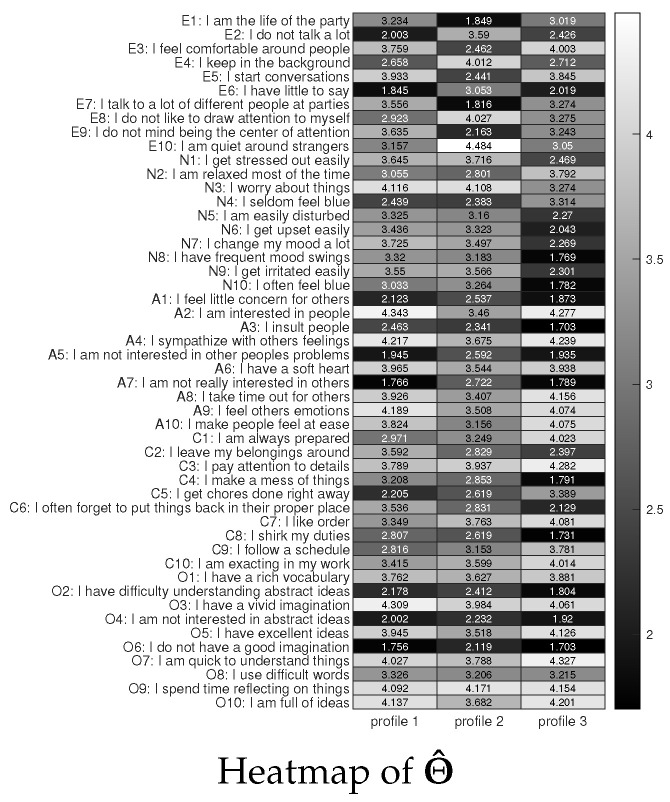
Numerical results for the BFPTRN data.

**Background**.Our SCK method is also applied to personality test data: the Big Five Personality Test with Random Number (BFPTRN) data. This dataset can be downloaded from the same URL as the IPIP data. This data asks respondents to generate random numbers in certain ranges attached to 50 personality items. The Big Five personality traits are extraversion (items E1–E10), neuroticism (items N1–N10), agreeableness (items A1–A10), conscientiousness (items C1–C10), and openness (items O1–O10). The original BFPTRN data contains 1369 subjects. After excluding subjects with missing responses or missing random numbers and removing those with random numbers exceeding the specified range, there remain 1155 subjects, i.e., N=1155,J=50. All items are rated using the same 5-point scale as the IPIP data, which results in $R\in {\left\{1,2,3,4,5\right\}}^{1155\times 50}$. The detail of each item and each range for random numbers can be found in Figure 11.

**Analysis.** The estimated number of extreme latent profiles for the BFPTRN dataset is 3. Applying the SCK approach to R with K=3 produces the 1155×3 matrix $\hat{Z}$ and the 50×3 matrix $\hat{\Theta }$. SCK takes around 1.6 s to process this data.

**Results.** Without confusion, we also let profile 1, profile 2, and profile 3 represent the three estimated extreme latent profiles. Profiles 1,2, and 3 have 409, 320, and 426 subjects, respectively. Similar to the IPIP data, based on $\hat{Z}$ and $\hat{\Theta }$, we can also obtain the heatmap of the average point on each subject for every profile, the heatmap of the average random number on each range for every profile, and the heatmap of $\hat{\Theta }$ as shown in Figure. We observe that there is no significant connection between the average point and the average random number on each item in each estimated extreme latent profile. From the first panel of Figure, we find that: for extroversion, subjects in profile 1 are the most extroverted, while subjects in profile 2 are the most introverted; for neuroticism, subjects in profile 3 are emotionally stable, while subjects in profiles 1 and 2 are emotionally unstable; for agreeableness, subjects in profiles 1 and 3 are easier to get along with than subjects in profile 2; for conscientiousness, subjects in profile 3 are more responsible than those in profiles 1 and 2; and for openness, subjects in profiles 1 and 3 are more open than those in profile 2. Meanwhile, the matrix shown in the first panel of Figure approximates $\hat{\Theta }$ well, which has been explained in Remark 6.

## 8. Conclusions and Future Work

In this paper, we introduced the arbitrary-distribution latent class model (adLCM), a novel class of latent class analysis models for data with arbitrary-distribution responses. We studied its model identifiability, developed an efficient inference method SCK to fit adLCM, and built a theoretical guarantee of estimation consistency for the proposed method under adLCM. On the methodology side, the new model adLCM provides exploratory and useful tools for latent class analysis in applications where the data may have arbitrary-distribution responses. adLCM allows the observed response matrix to be generated from any distribution as long as its expectation follows a latent class structure modeled by adLCM. In particular, the popular latent class model is a sub-model of our adLCM, and data with signed responses can also be modeled by adLCM. Ground-truth latent classes of data with responses generated from adLCM serve as benchmarks for evaluating latent class analysis approaches. On the algorithmic side, the SVD-based spectral method SCK is efficient and easy to implement. SCK requires no tuning parameters and it is applicable for data with arbitrary-distribution responses. This means that researchers in fields such as social, psychological, behavioral, and biological sciences, and beyond can design their tests/evaluations/surveys/interviews without worrying that the response should be binary or positive, as our method SCK is applicable for any kind of response matrices in latent class analysis. On the theoretic side, we established the rate of convergence for our method SCK under the proposed adLCM. We found that SCK exhibits different behaviors when the response matrices are generated from different distributions, and we conducted extensive experiments to verify our theoretical findings. Empirically, we applied our method to two real personality test datasets with meaningful results. We expect that our adLCM and SCK method will have broad applications for latent class analysis in understanding human behaviors for diverse fields, similar to the widespread use of latent class models in recent years.

There are several future directions worth exploring. First, methods with theoretical guarantees should be designed to determine the number of extreme latent profiles K for observed response matrices generated from any distribution F under adLCM. Following [81], a possible approach to estimate K is to count the number of significant singular values (i.e., those above a noise threshold) of R. Second, the grade of membership (GoM) model [52,82] provides a richer modeling capacity than the latent class model since GoM allows a subject to belong to multiple extreme latent profiles. Therefore, following the distribution-free idea developed in this work, it is meaningful to extend the model GoM to data with arbitrary-distribution responses. Third, like the LCM being equipped with individual covariates [50,83,84,85,86,87], it is worth considering adding individual covariates into the adLCM analysis. Fourth, our adLCM only considers static latent class analysis and it is meaningful to extend adLCM to the dynamic case [88]. Fifth, our SCK is a spectral clustering method, and it is possible to speed it up by application of the random-projection techniques [63] or the distributed spectral clustering idea [89] to deal with large-scale data for latent class analysis. Finally, while our proposed methods can estimate latent class memberships and item parameters, a meaningful extension would be to develop methodology and theoretical guarantees for explicitly estimating the latent distribution parameters in future work. 

## Figures and Tables

**Table 1:** Error rates of different methods for R in Figure 8. Values outside the parentheses correspond to the first panel of Figure 8, and those inside correspond to the second panel of Figure 8.

Method	Clustering	Hamming	NMI	ARI	Rel. $\ell_{1}$	Rel. $\ell_{2}$
	Error	Error			Error	Error
SCK	0 (0)	0 (0)	1.000 (1.000)	1.000 (1.000)	0.0024 (0.0254)	0.0032 (0.0295)
RMK	0 (0)	0 (0)	1.000 (1.000)	1.000 (1.000)	0.0024 (0.0245)	0.0032 (0.0295)
NMF	0 (0)	0 (0)	1.000 (1.000)	1.000 (1.000)	0.0024 (0.0245)	0.0032 (0.0295)
HC	0 (0)	0 (0)	1.000 (1.000)	1.000 (1.000)	0.0024 (0.0254)	0.0032 (0.0307)
PLCA	0 (0)	0 (0)	1.000 (1.000)	1.000 (1.000)	0.0024 (0.0245)	0.0032 (0.0295)

**Table 2:** Descriptive statistics for each estimated extreme latent profile from $\hat{Z}$ on the IPIP dataset.

Statistic	Profile 1	Profile 2	Profile 3
Size	276	226	394
# Male	123	129	241
# Female	153	97	153
Average age (male)	35.98	32.82	35.90
Average age (female)	35.54	31.38	38.71

## Data Availability

Data and code will be made available on request.

## Appendix A. Proofs Under adLCM

### Appendix A.1. Proof of Proposition 1

**Proof.** According to Lemma 1, we know that U=ZX, where $X=\Theta^{\prime }V\Sigma^{-1}$. Similarly, U can be rewritten as $U=\tilde{Z}\tilde{X}$, where $\tilde{X}={\tilde{\Theta }}^{\prime }V\Sigma^{-1}$. Then, for i∈[N], we have

(A1) $$\begin{array}{c} U\left(i,:\right)=Z\left(i,:\right)X=X\left(\ell \left(i\right),:\right)=\tilde{Z}\left(i,:\right)\tilde{X}=\tilde{X}\left(\tilde{\ell }\left(i\right),:\right), \end{array}$$

where $\tilde{\ell }\left(i\right)$ denotes the extreme latent profile that the i-th subject belongs in the alternative classification matrix $\tilde{Z}$. For $\bar{i}\in \left[N\right]$ and $\bar{i}\neq i$, we have

(A2) $$\begin{array}{c} U\left(\bar{i},:\right)=Z\left(\bar{i},:\right)X=X\left(\ell \left(\bar{i}\right),:\right)=\tilde{Z}\left(\bar{i},:\right)\tilde{X}=\tilde{X}\left(\tilde{\ell }\left(\bar{i}\right),:\right). \end{array}$$

When $\ell \left(i\right)=\ell \left(\bar{i}\right)$, by the second statement of Lemma 1, we get $U\left(i,:\right)=U\left(\bar{i},:\right)$. Combining this fact (i.e., $U\left(i,:\right)=U\left(\bar{i},:\right)$) with Equations (A1) and (A2) leads to

(A3) $$\begin{array}{c} X\left(\ell \left(i\right),:\right)=X\left(\ell \left(\bar{i}\right),:\right)=\tilde{X}\left(\tilde{\ell }\left(i\right),:\right)=\tilde{X}\left(\tilde{\ell }\left(\bar{i}\right),:\right)\;\mathrm{when}\;\ell \left(i\right)=\ell \left(\bar{i}\right). \end{array}$$

Equation (A3) implies that $\tilde{\ell }\left(i\right)=\tilde{\ell }\left(\bar{i}\right)$ when $\ell \left(i\right)=\ell \left(\bar{i}\right)$, i.e., for any two distinct subjects i and $\bar{i}$, they are in the same extreme latent profile under $\tilde{Z}$ when they are in the same extreme latent profile under Z. Therefore, we have $\tilde{Z}=ZP$, where P is a permutation matrix. Combining $\tilde{Z}=ZP$ with $Z\Theta^{\prime }=\tilde{Z}{\tilde{\Theta }}^{\prime }$ leads to $Z\Theta^{\prime }=\tilde{Z}{\tilde{\Theta }}^{\prime }=ZP{\tilde{\Theta }}^{\prime }$, which gives that

(A4) $$\begin{array}{c} Z(\Theta^{\prime }-P{\tilde{\Theta }}^{\prime })=0. \end{array}$$

Taking the transposition of Equation (A4) gives

(A5) $$\begin{array}{c} \left(\Theta -\tilde{\Theta }P^{\prime }\right)Z^{\prime }=0. \end{array}$$

Right multiplying Z at both sides of Equation (A6) gives

(A6) $$\begin{array}{c} \left(\Theta -\tilde{\Theta }P^{\prime }\right)Z^{\prime }Z=0. \end{array}$$

Since each extreme latent profile is not an empty set, the N×K classification matrix Z has a rank K, which gives that the K×K matrix $Z^{\prime }Z$ is nonsingular. Therefore, right multiplying ${\left(Z^{\prime }Z\right)}^{-1}$ at both sides of Equation (A6) gives $\Theta =\tilde{\Theta }P^{\prime }$, i.e., $\tilde{\Theta }=\Theta P$ since P is a permutation matrix. □

### Appendix A.2. Proof of Lemma 1

**Proof.** For the first statement: Since $R_{0}=Z\Theta^{\prime }=U\Sigma V^{\prime }$, $V^{\prime }V=I_{K_{0}\times K_{0}}$, and the $K_{0}\times K_{0}$ diagonal matrix Σ is nonsingular, we have $U=Z\Theta^{\prime }V\Sigma^{-1}\equiv \mathrm{ZX}$, where $X=\Theta^{\prime }V\Sigma^{-1}$. Hence, the first statement holds. For the second statement: For i∈[N], U=ZX gives U(i,:)=Z(i,:)X=X(ℓ(i),:). Then, if $\ell \left(\bar{i}\right)=\ell \left(i\right)$, we have $U\left(\bar{i},:\right)=X\left(\ell \left(\bar{i}\right),:\right)=X\left(\ell \left(i\right),:\right)=U\left(i,:\right)$, i.e., U has K distinct rows. Thus, the second statement holds. For the third statement: Since $R_{0}=Z\Theta^{\prime }=U\Sigma V^{\prime }$, we have $\Theta Z^{\prime }=V\Sigma U^{\prime }\Rightarrow \Theta Z^{\prime }Z=V\Sigma U^{\prime }Z\Rightarrow \Theta =V\Sigma U^{\prime }Z{\left(Z^{\prime }Z\right)}^{-1}$ where the K×K matrix $Z^{\prime }Z$ is nonsingular because each extreme latent profile has at least one subject, i.e., $\mathrm{rank}\left(Z^{\prime }Z\right)=\mathrm{rank}\left(Z\right)=K$. Thus, the third statement holds. For the fourth statement: Recall that when $K_{0}=K$, we have $U\in R^{N\times K},V\in R^{J\times K}$, Σ is a K×K full-rank diagonal matrix, and X is a K×K matrix, where $U^{\prime }U=I_{K\times K},V^{\prime }V=I_{K\times K}$. Let $\Delta =\mathrm{diag}(\sqrt{N_{1}},\sqrt{N_{2}},\ldots ,\sqrt{N_{K}})$, then

(A7) $$\begin{array}{c} R_{0}=Z\Theta^{\prime }=Z\Delta^{-1}\Delta \Theta^{\prime }. \end{array}$$

It is straightforward to verify that $Z\Delta^{-1}$ is a column orthogonal matrix, i.e., ${\left(Z\Delta^{-1}\right)}^{\prime }Z\Delta^{-1}=I_{K\times K}$. Since $K_{0}=K$, we have $\mathrm{rank}(\Delta \Theta^{\prime })=K$. Let $\tilde{U}\tilde{\Sigma }{\tilde{V}}^{\prime }=\Delta \Theta^{\prime }$ be the compact SVD of $\Delta \Theta^{\prime }$, where $\tilde{\Sigma }$ is a K×K diagonal matrix, $\tilde{U}\in R^{K\times K},\tilde{V}\in R^{J\times K},{\tilde{U}}^{\prime }\tilde{U}=I_{K\times K}$, and ${\tilde{V}}^{\prime }\tilde{V}=I_{K\times K}$. Note that $\tilde{U}\in R^{K\times K}$ and ${\tilde{U}}^{\prime }\tilde{U}=I_{K\times K}$ imply $\mathrm{rank}(\tilde{U})=K$. Equation (A7) implies

(A8) $$\begin{array}{c} R_{0}=Z\Theta^{\prime }=U\Sigma V^{\prime }=Z\Delta^{-1}\Delta \Theta^{\prime }=Z\Delta^{-1}\tilde{U}\tilde{\Sigma }{\tilde{V}}^{\prime }. \end{array}$$

Note that $U,V,Z\Delta^{-1}\tilde{U}$, and $\tilde{V}$ are all orthonormal matrices. Moreover, note that Σ and $\tilde{\Sigma }$ are K×K diagonal matrices. Then we have

(A9) $$\begin{array}{c} U=Z\Delta^{-1}\tilde{U},\Sigma =\tilde{\Sigma },\mathrm{and}\;V=\tilde{V}. \end{array}$$

Recall that U=ZX; Equation (A9) gives that $X=\Delta^{-1}\tilde{U}\in R^{K\times K}$ and rank(X)=K because rank(Δ)=K and $\mathrm{rank}(\tilde{U})=K$. We can easily verify that the rows of $\Delta^{-1}\tilde{U}$ are perpendicular to each other and the k-th row has length $\sqrt{1/N_{k}}$ for k∈[K], i.e., $\sqrt{{\mathrm{XX}}^{\prime }}=\sqrt{\Delta^{-1}\tilde{U}{\tilde{U}}^{\prime }\Delta^{-1}}=\sqrt{\Delta^{-2}}=\Delta^{-1}$. Thus, the fourth statement holds.
**Remark** **A1.** *In this remark, we provide the reason why the fourth statement does not hold when $K_{0}<K$. For this case, the rank of $\Delta \Theta^{\prime }$ is $K_{0}$, and thus $\tilde{U}\in R^{K\times K_{0}}$ and $\mathrm{rank}\left(\tilde{U}\right)=K_{0}$. Then we have $X=\Delta^{-1}\tilde{U}\in R^{K\times K_{0}}$ and $\mathrm{rank}\left(X\right)=K_{0}$. Thus, $\mathrm{rank}\left({\mathrm{XX}}^{\prime }\right)=K_{0}<K=\mathrm{rank}\left(\Delta^{-2}\right)$, which implies $\sqrt{{\mathrm{XX}}^{\prime }}\neq \Delta^{-1}$ when $K_{0}<K$ and the fourth statement does not hold.*
□

### Appendix A.3. Proof of Theorem 1

First, the following two lemmas are provided for our further proof.

**Lemma** **A1.**
*Under adLCM(Z,Θ,F), we have*

$$\begin{array}{c} \max(∥\hat{U}\hat{O}{-U∥}_{F},∥\hat{V}\hat{O}-V{∥}_{F})\leq \frac{2\sqrt{2K}∥R-R_{0}∥}{\rho \sigma_{K}\left(B\right)\sqrt{N_{\min}}}, \end{array}$$

*where $\hat{O}$ is a K-by-K orthogonal matrix.*

**Proof.** According to the proof of Lemma 3 in [62], there is a K×K orthogonal matrix $\hat{O}$ such that

$$\begin{array}{c} \max(∥\hat{U}\hat{O}{-U∥}_{F},∥\hat{V}\hat{O}-V{∥}_{F})\leq \frac{\sqrt{2K}∥\hat{R}-R_{0}∥}{\sqrt{\lambda_{K}\left(R_{0}R_{0}^{\prime }\right)}}. \end{array}$$

Because $\hat{R}$ is the top K SVD of R and $\mathrm{rank}(R_{0})=K$, we have $∥R-\hat{R}∥\leq ∥R-R_{0}∥$. Then we have $∥\hat{R}-R_{0}∥=∥\hat{R}-R+R-R_{0}∥\leq 2∥R-R_{0}∥$, which gives

(A10) $$\begin{array}{c} \max(∥\hat{U}\hat{O}{-U∥}_{F},∥\hat{V}\hat{O}-V{∥}_{F})\leq \frac{2\sqrt{2K}∥R-R_{0}∥}{\sqrt{\lambda_{K}\left(R_{0}R_{0}^{\prime }\right)}}. \end{array}$$

For $\lambda_{K}\left(R_{0}R_{0}^{\prime }\right)$, because $R_{0}=Z\Theta^{\prime }=\rho {\mathrm{ZB}}^{\prime }$ and $\lambda_{K}\left(Z^{\prime }Z\right)=N_{\min}$, we have

$$\begin{array}{cc} \lambda_{K}\left(R_{0}R_{0}^{\prime }\right) & =\lambda_{K}\left(Z\Theta^{\prime }\Theta Z^{\prime }\right)=\lambda_{K}\left(\rho^{2}{\mathrm{ZB}}^{\prime }{\mathrm{BZ}}^{\prime }\right)=\rho^{2}\lambda_{K}\left(B^{\prime }{\mathrm{BZ}}^{\prime }Z\right)\\ & \geq \rho^{2}\lambda_{K}\left(Z^{\prime }Z\right)\lambda_{K}\left(B^{\prime }B\right)=\rho^{2}N_{\min}\lambda_{K}\left(B^{\prime }B\right). \end{array}$$

Combining Equation (A10) with $\lambda_{K}\left(R_{0}R_{0}^{\prime }\right)\geq \rho^{2}N_{\min}\lambda_{K}\left(B^{\prime }B\right)$ gives

$$\begin{array}{c} \max(∥\hat{U}\hat{O}{-U∥}_{F},∥\hat{V}\hat{O}-V{∥}_{F})\leq \frac{2\sqrt{2K}∥R-R_{0}∥}{\rho \sigma_{K}\left(B\right)\sqrt{N_{\min}}}. \end{array}$$

□

**Lemma** **A2.**
*Under adLCM(Z,Θ,F), if Assumption 1 is satisfied, then with a probability of at least $1-o({\left(N+J\right)}^{-3})$,*

$$\begin{array}{c} ∥R-R_{0}∥\leq C\sqrt{\gamma \;\max(N,J)\log(N+J)}, \end{array}$$

*where C is a positive constant.*

**Proof.** This lemma holds by setting  α in Lemma 2 [90] as 3, where Lemma 2 of [90] is obtained from the rectangular version of Bernstein inequality in [91]. □

**Proof.** Now, we prove the first statement of Theorem 1. Set ς>0 as a small value, by Lemma 2 of [61] and the fourth statement of Lemma 1, if

(A11) $$\begin{array}{c} \frac{\sqrt{K}}{\varsigma }{∥U-\hat{U}\hat{O}∥}_{F}\left(\frac{1}{\sqrt{N_{k}}}+\frac{1}{\sqrt{N_{l}}}\right)\leq \sqrt{\frac{1}{N_{k}}+\frac{1}{N_{l}}},\;\mathrm{for}\;\mathrm{each}\;1\leq k\neq l\leq K, \end{array}$$

and then the Clustering error $\hat{f}=O\left(\varsigma^{2}\right)$ using the K-means algorithm. By setting $\varsigma \;=\;\sqrt{\frac{2KN_{\max}}{N_{\min}}}{∥U-\hat{U}\hat{O}∥}_{F}$, we see that Equation (A11) always holds for all 1≤k≠l≤K. Thus we get $\hat{f}=O\left(\varsigma^{2}\right)=O\left(\frac{KN_{\max}{∥U-\hat{U}\hat{O}∥}_{F}^{2}}{N_{\min}}\right)$. According to Lemma A1, we have

$$\begin{array}{c} \hat{f}=O\left(\frac{K^{2}N_{\max}{∥R-R_{0}∥}^{2}}{\rho^{2}\sigma_{K}^{2}\left(B\right)N_{\min}^{2}}\right). \end{array}$$

By Lemma A2, we have

$$\begin{array}{c} \hat{f}=O\left(\frac{\gamma K^{2}N_{\max}\max\left(N,J\right)\log\left(N+J\right)}{\rho^{2}\sigma_{K}^{2}\left(B\right)N_{\min}^{2}}\right). \end{array}$$

Next, we prove the second statement of Theorem 1. Since U=ZX by Equation (3) in Lemma 1 and $U^{\prime }U=I_{K\times K}$, we have $X^{\prime }Z^{\prime }\mathrm{ZX}=I_{K\times K}$ which gives that ${\left(Z^{\prime }Z\right)}^{-1}={\mathrm{XX}}^{\prime }$ and $\lambda_{1}\left({\mathrm{XX}}^{\prime }\right)=\sigma_{1}^{2}\left({\mathrm{XX}}^{\prime }\right)=\frac{1}{\lambda_{K}\left(Z^{\prime }Z\right)}=\frac{1}{N_{\min}}$. We also have $Z{\left(Z^{\prime }Z\right)}^{-1}={\mathrm{ZXX}}^{\prime }={\mathrm{UX}}^{\prime }$. Similarly, we have $\hat{Z}{\left({\hat{Z}}^{\prime }\hat{Z}\right)}^{-1}\approx \hat{U}{\hat{X}}^{\prime }$, where $\hat{X}$ is the K×K centroid matrix returned by the K-means method for $\hat{U}$. Recall that $\hat{R}=\hat{U}\hat{\Sigma }{\hat{V}}^{\prime }$, then combine it with Equation (4) and Lemma A2, and we have

$$\begin{array}{cc} ∥\hat{\Theta }-\Theta P∥ & =∥\hat{V}\hat{\Sigma }{\hat{U}}^{\prime }\hat{Z}{\left({\hat{Z}}^{\prime }\hat{Z}\right)}^{-1}-V\Sigma U^{\prime }Z{\left(Z^{\prime }Z\right)}^{-1}P∥=∥{\hat{R}}^{\prime }\hat{Z}{\left({\hat{Z}}^{\prime }\hat{Z}\right)}^{-1}-R_{0}^{\prime }Z{\left(Z^{\prime }Z\right)}^{-1}P∥\\ \; & =∥\left({\hat{R}}^{\prime }-R_{0}^{\prime }\right)\hat{Z}{\left({\hat{Z}}^{\prime }\hat{Z}\right)}^{-1}+R_{0}^{\prime }\left(\hat{Z}{\left({\hat{Z}}^{\prime }\hat{Z}\right)}^{-1}-Z{\left(Z^{\prime }Z\right)}^{-1}P\right)∥\\ \; & \leq ∥\left({\hat{R}}^{\prime }-R_{0}^{\prime }\right)\hat{Z}{\left({\hat{Z}}^{\prime }\hat{Z}\right)}^{-1}∥+∥R_{0}^{\prime }\left(\hat{Z}{\left({\hat{Z}}^{\prime }\hat{Z}\right)}^{-1}-Z{\left(Z^{\prime }Z\right)}^{-1}P\right)∥\\ \; & \leq ∥\hat{R}-R_{0}∥∥\hat{Z}{\left({\hat{Z}}^{\prime }\hat{Z}\right)}^{-1}∥+∥R_{0}∥\hat{Z}{\left({\hat{Z}}^{\prime }\hat{Z}\right)}^{-1}-Z{\left(Z^{\prime }Z\right)}^{-1}P∥\\ \; & \leq 2∥R-R_{0}∥∥\hat{Z}{\left({\hat{Z}}^{\prime }\hat{Z}\right)}^{-1}∥+∥R_{0}∥\hat{Z}{\left({\hat{Z}}^{\prime }\hat{Z}\right)}^{-1}-Z{\left(Z^{\prime }Z\right)}^{-1}P∥\\ \; & =2∥R-R_{0}∥∥\hat{Z}{\left({\hat{Z}}^{\prime }\hat{Z}\right)}^{-1}∥+∥\rho {\mathrm{ZB}}^{\prime }∥∥\hat{Z}{\left({\hat{Z}}^{\prime }\hat{Z}\right)}^{-1}-Z{\left(Z^{\prime }Z\right)}^{-1}P∥\\ \; & \leq 2∥R-R_{0}∥∥\hat{Z}{\left({\hat{Z}}^{\prime }\hat{Z}\right)}^{-1}∥+\rho ∥Z∥∥B∥∥\hat{Z}{\left({\hat{Z}}^{\prime }\hat{Z}\right)}^{-1}-Z{\left(Z^{\prime }Z\right)}^{-1}P∥\\ \; & =2∥R-R_{0}∥∥\hat{Z}{\left({\hat{Z}}^{\prime }\hat{Z}\right)}^{-1}∥+\\ \; & \;\;\;\;\;\rho \sigma_{1}\left(B\right)\sqrt{N_{\max}}∥\hat{Z}{\left({\hat{Z}}^{\prime }\hat{Z}\right)}^{-1}-Z{\left(Z^{\prime }Z\right)}^{-1}P∥\\ \; & =2∥R-R_{0}∥∥\hat{Z}{\left({\hat{Z}}^{\prime }\hat{Z}\right)}^{-1}-Z{\left(Z^{\prime }Z\right)}^{-1}P+Z{\left(Z^{\prime }Z\right)}^{-1}P∥+\\ \; & \;\;\;\;\;\rho \sigma_{1}\left(B\right)\sqrt{N_{\max}}∥\hat{Z}{\left({\hat{Z}}^{\prime }\hat{Z}\right)}^{-1}-Z{\left(Z^{\prime }Z\right)}^{-1}P∥\\ \; & \leq 2∥R-R_{0}∥(∥\hat{Z}{\left({\hat{Z}}^{\prime }\hat{Z}\right)}^{-1}-Z{\left(Z^{\prime }Z\right)}^{-1}P∥+∥Z{\left(Z^{\prime }Z\right)}^{-1}P∥)+\\ \; & \;\;\;\;\;\rho \sigma_{1}\left(B\right)\sqrt{N_{\max}}∥\hat{Z}{\left({\hat{Z}}^{\prime }\hat{Z}\right)}^{-1}-Z{\left(Z^{\prime }Z\right)}^{-1}P∥\\ \; & \leq 2∥R-R_{0}∥(∥\hat{Z}{\left({\hat{Z}}^{\prime }\hat{Z}\right)}^{-1}-Z{\left(Z^{\prime }Z\right)}^{-1}P∥+∥Z{\left(Z^{\prime }Z\right)}^{-1}∥∥P∥)+\\ \; & \;\;\;\;\;\rho \sigma_{1}\left(B\right)\sqrt{N_{\max}}∥\hat{Z}{\left({\hat{Z}}^{\prime }\hat{Z}\right)}^{-1}-Z{\left(Z^{\prime }Z\right)}^{-1}P∥\\ \; & =2∥R-R_{0}∥(∥\hat{Z}{\left({\hat{Z}}^{\prime }\hat{Z}\right)}^{-1}-Z{\left(Z^{\prime }Z\right)}^{-1}P∥+\frac{1}{\sqrt{N_{\min}}})+\\ \; & \;\;\;\;\;\rho \sigma_{1}\left(B\right)\sqrt{N_{\max}}∥\hat{Z}{\left({\hat{Z}}^{\prime }\hat{Z}\right)}^{-1}-Z{\left(Z^{\prime }Z\right)}^{-1}P∥\\ \; & =O(∥R-R_{0}∥(∥\hat{U}{\hat{X}}^{\prime }-{\mathrm{UX}}^{\prime }P∥+\frac{1}{\sqrt{N_{\min}}}))\\ \; & \leq O(∥R-R_{0}∥(∥\hat{U}{\hat{X}}^{\prime }∥+∥{\mathrm{UX}}^{\prime }P∥+\frac{1}{\sqrt{N_{\min}}}))\\ \; & \leq O(∥R-R_{0}∥(∥\hat{U}∥∥\hat{X}∥+∥U∥∥X∥∥P∥+\frac{1}{\sqrt{N_{\min}}}))\\ \; & =O(∥R-R_{0}∥(∥\hat{X}∥+∥X∥+\frac{1}{\sqrt{N_{\min}}}))\\ \; & =O(\frac{∥R-R_{0}∥}{\sqrt{N_{\min}}})\\ \; & =O(\sqrt{\frac{\gamma \;\max(N,J)\log(N+J)}{N_{\min}}}) \end{array}$$

Since $\left(\hat{\Theta }-\Theta P\right)$ is a J×K matrix and K≪J in this paper, we have $\mathrm{rank}(\hat{\Theta }-\Theta P)=K$. Since ${∥M∥}_{F}\leq \sqrt{\mathrm{rank}(M)}∥M∥$ holds for any matrix M, we have $∥\hat{\Theta }{-\Theta P∥}_{F}\leq \sqrt{K}∥\hat{\Theta }-\Theta P∥$. Thus, we have

(A12) $$\begin{array}{c} ∥\hat{\Theta }{-\Theta P∥}_{F}=O\left(\sqrt{\frac{\gamma K\;\max(N,J)\log(N+J)}{N_{\min}}}\right). \end{array}$$

Combining Equation (A12) with the fact that ${∥\Theta ∥}_{F}\geq ∥\Theta ∥=∥\rho B∥=\rho ∥B∥=\rho \sigma_{1}\left(B\right)\geq \rho \sigma_{K}\left(B\right)$ gives

$$\begin{array}{c} \frac{∥\hat{\Theta }{-\Theta P∥}_{F}}{{∥\Theta ∥}_{F}}\leq \frac{∥\hat{\Theta }{-\Theta P∥}_{F}}{\rho \sigma_{K}\left(B\right)}=O\left(\frac{\sqrt{\gamma K\max(N,J)\log(N+J)}}{\rho \sigma_{K}\left(B\right)\sqrt{N_{\min}}}\right). \end{array}$$

Recall that the J-by-K matrix B satisfies $\max_{j\in [J],k\in [K]}\left|B\left(j,k\right)\right|=1$; we have $\sigma_{K}\left(B\right)$ is at least of the order $\sqrt{J}-\sqrt{K-1}$ with high probability by applying the lower bound of the smallest singular value of a random rectangular matrix in [92]. Since K≪J in this paper, we have

$$\begin{array}{cc} \; & \hat{f}=O\left(\frac{\gamma K^{2}N_{\max}\max\left(N,J\right)\log\left(N+J\right)}{\rho^{2}N_{\min}^{2}J}\right)\;\mathrm{and}\;\frac{∥\hat{\Theta }{-\Theta P∥}_{F}}{{∥\Theta ∥}_{F}}=O\left(\frac{\sqrt{\gamma K\max(N,J)\log(N+J)}}{\rho \sqrt{N_{\min}J}}\right). \end{array}$$

□
